# *Helicobacter pylori* modulates host cell responses by CagT4SS-dependent translocation of an intermediate metabolite of LPS inner core heptose biosynthesis

**DOI:** 10.1371/journal.ppat.1006514

**Published:** 2017-07-17

**Authors:** Saskia C. Stein, Eugenia Faber, Simon H. Bats, Tatiana Murillo, Yvonne Speidel, Nina Coombs, Christine Josenhans

**Affiliations:** 1 Medizinische Hochschule Hannover, Institute for Medical Microbiology, Hannover, Germany; 2 German Center for Infection Research (DZIF), partner site Hannover-Braunschweig, Hannover, Germany; Stanford University School of Medicine, UNITED STATES

## Abstract

Highly virulent *Helicobacter pylori* cause proinflammatory signaling inducing the transcriptional activation and secretion of cytokines such as IL-8 in epithelial cells. Responsible in part for this signaling is the *cag* pathogenicity island (*cag*PAI) that codetermines the risk for pathological sequelae of an *H*. *pylori* infection such as gastric cancer. The Cag type IV secretion system (CagT4SS), encoded on the *cag*PAI, can translocate various molecules into cells, the effector protein CagA, peptidoglycan metabolites and DNA. Although these transported molecules are known to contribute to cellular responses to some extent, a major part of the *cag*PAI-induced signaling leading to IL-8 secretion remains unexplained. We report here that biosynthesis of heptose-1,7-bisphosphate (HBP), an important intermediate metabolite of LPS inner heptose core, contributes in a major way to the *H*. *pylori cag*PAI-dependent induction of proinflammatory signaling and IL-8 secretion in human epithelial cells. Mutants defective in the genes required for synthesis of HBP exhibited a more than 95% reduction of IL-8 induction and impaired CagT4SS-dependent cellular signaling. The loss of HBP biosynthesis did not abolish the ability to translocate CagA. The human cellular adaptor TIFA, which was described before to mediate HBP-dependent activity in other Gram-negative bacteria, was crucial in the *cag*PAI- and HBP pathway-induced responses by *H*. *pylori* in different cell types. The active metabolite was present in *H*. *pylori* lysates but not enriched in bacterial supernatants. These novel results advance our mechanistic understanding of *H*. *pylori cag*PAI-dependent signaling mediated by intracellular pattern recognition receptors. They will also allow to better dissect immunomodulatory activities by *H*. *pylori* and to improve the possibilities of intervention in *cagPAI-* and inflammation-driven cancerogenesis.

## Author summary

The Cag Type IV secretion system, which contributes to inflammation and cancerogenesis during chronic infection, is one of the major virulence and fitness factors of the bacterial gastric pathogen *Helicobacter pylori*. Up to now, the mechanisms leading to *cag*PAI-dependent signal transduction and cytokine secretion were not completely understood. We report here that HBP, an intermediate metabolite in LPS core heptose biosynthesis, is translocated into host cells dependent on the CagT4SS, and is a major factor leading to the activation of cellular responses. This response is connected to the human cellular adaptor protein TIFA. The knowledge of this specific response pathway is a major advance in understanding CagT4SS-dependent signaling and will enable us to understand better how *H*. *pylori* modulates the immune response repertoire in its human host.

## Introduction

The successful bacterial gastric colonizer and human pathogen *Helicobacter pylori* has conquered a largely uninhabitable niche, the human stomach mucosa, where it persists in about 50% of the world’s population. Navigation systems guide the bacteria closely to the gastric mucosa, where they dwell in gastric crypts [1–3] and along the mucosal surface in a deep layer of the stomach mucus which can reach close to neutral pH [4–6]. The intimate association with host epithelium seems to be necessary for the pathogen to maintain persistent colonization. Its coevolution with the human host for more than 50,000 years [7–10] has enabled the bacteria to gain a foothold in the stomach epithelial niche against mucus shedding, the continuously changing environmental conditions and individual human physiology and immune responses. Not only the changing niche but also the individual host phenotypes require that the bacterial host interaction repertoire adjusts itself upon each transmission event and also during a lifetime of persistence in one evolving host individual [11].

To achieve this purpose in an unusually efficient manner, *H*. *pylori* has developed several arms of modulating its host interactions: within the stomach and upon transmission, *H*. *pylori* can change its genetic repertoire by a high mutation rate [12] and an even higher propensity to recombine and shuffle its DNA during mixed infection [13]. Its distinct and variable expression of immunomodulatory and immune evasive mechanisms stands out as a third arm of host modulation. Generally speaking, *H*. *pylori* has evolved to be a rather immune evasive organism. First of all, this is strikingly demonstrated by a lower propensity of classical MAMPs of the organisms, such as their lipid A and lipopolysaccharide (LPS) [4–16] and flagellins [17–19], to elicit host immune activation. *H*. *pylori* lipid A has specific modifications and is hypoacylated, which is responsible for its low activity as a TLR4 agonist [20;21]. Although *H*. *pylori* MAMPs have retained, albeit lower, activity for TLR stimulation, and *H*. *pylori* can activate cells via TLR and inflammasomes [22–24], the most prominent driver of host responses is the *cag* pathogenicity island (*cag*PAI). Approximately 70% of all *H*. *pylori* worldwide possess the island [8]. This 37 kb genomic island harbours genes which can form a type 4 secretion system (T4SS) apparatus [8;25–27]. The CagT4SS can make contact with host cells, for instance via integrins [28–31], and was reported to influence host cell signaling in multiple ways [32–34], dependent on intimate bacteria-cell contact. The *cag*PAI displays substantial genetic variation between strains from different geographical regions [8], which offers a rich repertoire for generating recombinatorial variants. Even within one host stomach, *cag*PAI genes, the expression of *cag*PAI components or *cag*PAI-dependent cell activation phenotypes can vary. Examples for this variation have been identified in CagA and CagY [35;36].

The *cag*PAI serves the bacteria as a major modulator of host cell responses. Its presence is crucial to instigate proinflammatory signaling in the host, for instance leading to the activation of NF-κB and other transcription factors, and to the secretion of proinflammatory cytokines and chemokines such as IL-8 [37]. It is also associated with the acquisition of iron inside the host stomach [38]. This latter capacity is linked to the expression of CagA, an oncogenic protein encoded on the *cag*PAI [39;40]. CagA is transported and inserted as an effector protein into host cells by the CagT4SS [41;42]. CagA only minimally contributes to IL-8 secretion [34;43], but appears to be involved in NF-κB signaling and interacts with different host pathways mediated by SH-1 and SH-2 domains and phosphorylation-dependent signal transduction [44]. The inter-strain variability of the CagA effector is high [8], and the bacteria carry CagA in different sequence variants that can be linked to host ethnicity [32;45], for instance to Western and Eastern human populations. Other bacterial molecules that the T4SS translocates into host cells are peptidoglycan metabolites [46] and bacterial DNA [47].

It is well established that the *cag*PAI is crucial for the strong proinflammatory signals that *H*. *pylori* elicits in host cells and the infected stomach, enhancing chronic gastric inflammation. However, questions remain with regard to the exact nature of the signals and the sequence of events contributing to the strong and rapid innate response. Peptidoglycan biosynthesis metabolites, for instance gamma-D-glutamyl-meso-diaminopimelic acid (iE-DAP), appear to contribute via CagT4SS-dependent, NOD1-mediated signal transduction [46]. The cellular signaling cascade addressed in gastric epithelial cells *cag*PAI-dependently by *H*. *pylori* involves TRAF6, canonical and non-canonical NF-κB activation and TAK1 phosphorylation [48–51].

Earlier reports stated that the multifaceted nature and variable modifications of *H*. *pylori* LPS, such as phosphorylation status [52–54], contribute to the strength of the signal during *H*. *pylori*-host interaction. Genes involved in Lipid A and LPS outer chain biosynthesis and modification have been characterized to rather mediate immunoevasive properties [54;55] and appeared to offer a minor contribution in proinflammatory cell activation by *H*. *pylori* [21]. On the other hand, numerous genes of core LPS biosynthesis were ruled out for a mutagenesis approach, since various fundamental LPS genes including those of lipid A biosynthesis were essential for the viability and survival of *H*. *pylori* in the laboratory setting even in vitro [56;57].

Therefore, we set out in the present study to investigate the contribution of the first genes of LPS inner core heptose biosynthesis and its potential metabolites to the immediate early innate signaling induced by *H*. *pylori* in human cells. We also took into account recent findings that other bacterial species may activate cells via an intermediate heptose of LPS inner core heptose biosynthesis [58]. We demonstrate here that active *H*. *pylori* LPS core heptose synthesis producing the metabolite heptose-1,7-bisphosphate (HBP) contributes to *cag*PAI-dependent signaling and proinflammatory cytokine release in human epithelial cells in a major way, and that the human cellular adaptor protein TIFA is essential for this response.

## Results

### *H*. *pylori*-driven IL-8 secretion in human gastric epithelial cells is dependent on LPS inner core heptose biosynthesis pathways

Since previous studies have indicated a multifactorial role of *H*. *pylori* LPS in immune activation and modulation [52;53], we performed a mutagenesis of LPS inner core heptose biosynthesis-related genes of *H*. *pylori*. We focussed on the first genes of LPS inner core heptose biosynthesis [59;60], which, in *H*. *pylori* as in other Gram-negative bacteria, may form the basic building blocks for the attachment of heptoses to the LPS ketodeoxy-octonate (KDO) core by heptose transferases such as WaaC (HP0279; [61;62]) and for the subsequent biosynthesis of LPS outer core and outer chains (smooth LPS).

**Fig 1:**
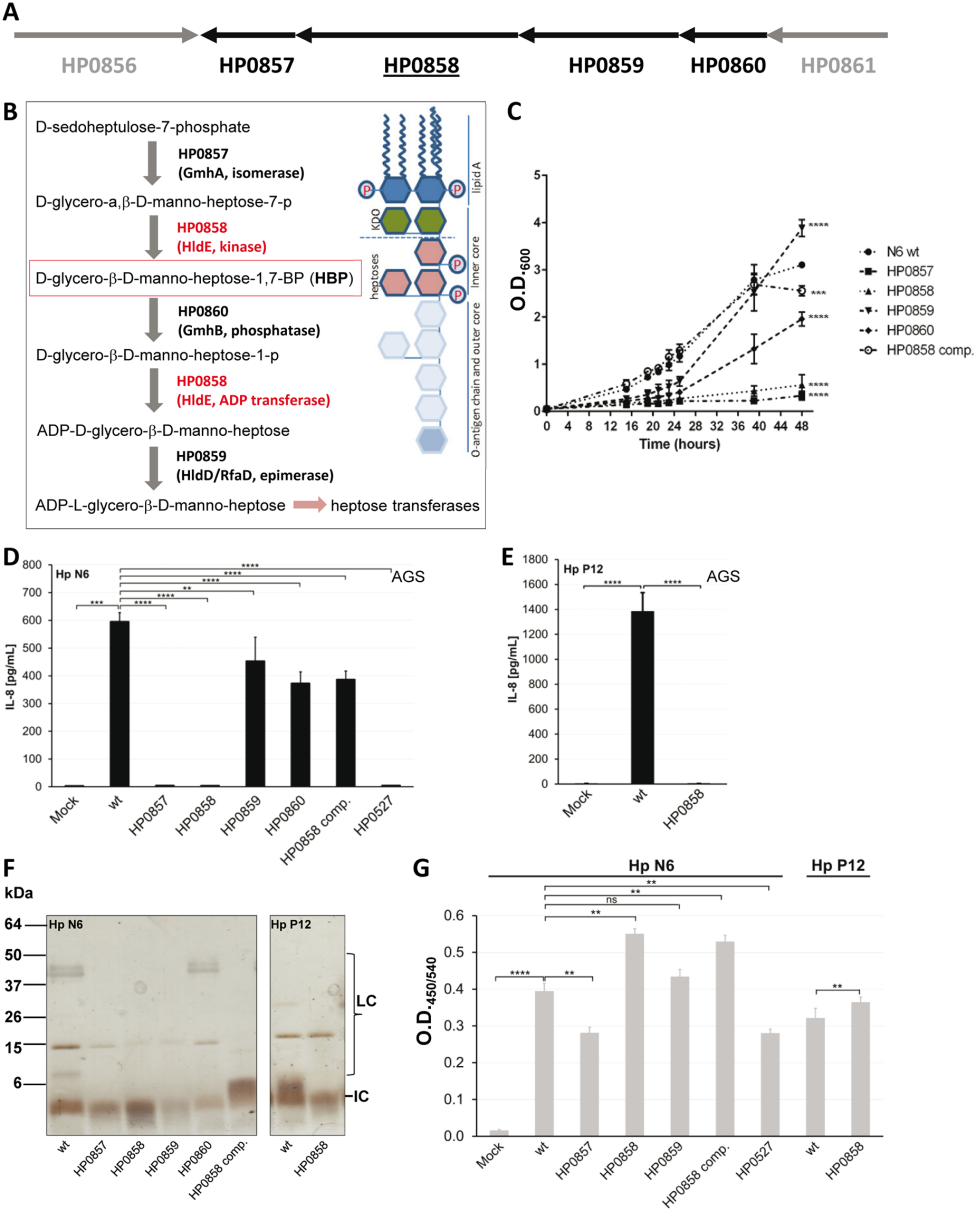
The *H*. *pylori hldE* (core LPS heptose biosynthesis) gene cluster, its proposed biosynthetic pathway, and strain characterization of *H*. *pylori hldE* gene cluster mutants. **A**) Heptose phosphate biosynthesis gene cluster in *H*. *pylori*. Genes HP0861 through HP0857 of reference strain 26695 probably form an operon (see also [110]). **B**) Biosynthesis pathway, LPS intermediates, metabolites and contributing enzymes of the core heptose pathway predicted in *H*. *pylori*. In red color, the biosynthesis functions of the bifunctional *hldE* gene (HP0858) in analogy to its *E*. *coli* ortholog are depicted within the pathway. The metabolite HBP, produced through the action of HldE enzyme, is highlighted by a red square. To the right, a schematic structure of *H*. *pylori* LPS is shown, indicating its main structural building blocks. The dashed line indicates the potential intermediate LPS structure in the absence of the GmhA/HldE pathway, which consists solely of the lipid A and the keto-deoxy-octonate (KDO) substructures. Gene designations refer to gene names in strain 26695. For references regarding LPS biosynthesis see main text. **C**) Growth characteristics of *H*. *pylori* strain N6 core heptose phosphate biosynthesis mutants (designated by strain 26695 gene numbers) in comparison to the N6 reference strain. Growth curves of single strains were performed in liquid culture for up to 48 h. **D**), **E**) Quantification of IL-8 secretion of AGS cells coincubated for 4 h with live *H*. *pylori* strains. Hp N6 = strain N6 shown in panels **D**, **F**, **G**; Hp P12 = strain P12 shown in panels **E**,**F**,**G**. **F**) silver-stained SDS gel of treated lysate (ETL) preparations (Methods) from *H*. *pylori* and its core heptose biosynthesis mutants. Detected glycans include *H*. *pylori* LPS chains as annotated on the right: IC (inner core of LPS), LC (longer LPS chains, including outer core and O-antigen chains) [16] **G**) Determination of cell adherence of *H*. *pylori* wild type strains N6 and P12 and their isogenic core heptose biosynthesis mutants to AGS cells (Methods). Strain designations in all figure panels refer to gene names in the *gmhA/hldE* cluster of strain 26695 as indicated in panel B. comp. = complemented. Statistically significant differences between strains during growth in liquid culture in panel **C** were calculated by two-way ANOVA, followed by Tukey’s multiple comparison test: **p<0.01, ****p<0.0001, they are depicted next to the graphs for the last time point (48 h); see also S1 Table for full results. Statistical significances in **D)**, **E), G)** were determined by two-tailed, non-paired Student’s *t*-test; **p<0.01, ***p<0.001, ****p<0.0001, ns = non-significant.

We generated and tested mutants in *H*. *pylori* genes *gmhA* (HP0857), *hldE* (*rfaE*; HP0858), *rfaD* (HP0859), and *gmhB* (HP0860), which form a contiguous gene cluster which encodes interlinked enzymatic functions (Fig 1A and 1B), in the *cag*PAI-positive strain N6 [63]. HP0858 mutants were also obtained in a second, well-characterized strain, P12 [64] which likewise expresses an active CagT4SS. Despite the fact that the complete gene cluster of the core heptose pathway (Fig 1A) including the *hldE* coding sequences (S1 Fig) are invariably present in a global selection of *H*. *pylori* genomes, generating mutants in HP0858 and HP0859 orthologs was not possible in several other tested strains. We verified the strain-specific essential function of HP0858 by introducing a second copy of the HP0858 gene into the *rdxA* locus in *H*. *pylori* strain SU2 [8], which was not amenable to HP0858 insertion mutagenesis. Subsequently, it was possible in this strain to disrupt either one of the HP0858 gene copies. These findings underlined the partially strain-specific essential function of HP0858, which is likely to influence multiple properties of the bacterial envelope in *H*. *pylori*. We tested growth in vitro (Fig 1C), bacterial morphology by transmission electron microscopy and live-dead-stain fluorescence microscopy (S2 and S3 Figs), and antibiotic susceptibility (S2E Fig) of the LPS heptose core mutants in comparison to the parental strain. The mutants of the cluster had growth defects in liquid cultures to a different extent (Fig 1C); all mutants showed attenuated growth in comparison to the parent. Only the HP0859 mutant was able to reproducibly overcome an initial growth delay (Fig 1C). The HP0858 mutant also displayed an increased susceptibility to antibiotics (rifampicin, tetracyclin) in comparison to the parental strain (S2E Fig). Moreover, the gene insertion mutations led to the appearance of “rough” non-glossy colony growth of the bacteria on blood agar plates, in contrast to the commonly glossy colonies of the parental bacteria that produce smooth LPS. In order to address the question whether outer membrane integrity and protein composition were impaired in the mutants, we prepared whole cell lysates. Major outer membrane proteins (HopZ and BabB) and CagPAI proteins remained detectable in the core heptose mutants (S2F Fig), although the HP0857 and HP0858 mutants showed a clear decrease in amounts of CagA and CagL and an inverse alteration in outer membrane proteins HopZ and BabB (S2F and S2G Fig). While the mutants exhibited no major differences in single cell morphology upon growth on blood plates (S2 Fig, panels A-D), they tended to be more aggregative and showed variable differences in cell morphology such as shortened or elongated cells upon growth in liquid culture (S3 Fig).

All mutants were then tested on human gastric epithelial cells (AGS) for their ability to induce cytokine interleukin-8 (IL-8) release, which is one of the hallmarks of *cag*PAI-dependent signaling in host cells. While all clones of the HP0860 and HP0859 mutants were able to significantly activate IL-8 secretion, albeit less than the parental strain (Fig 1D), the HP0857 and HP0858 mutants (four clones of each tested) were all completely deficient in IL-8 induction (Fig 1D and 1E for HP0858 mutant of strain P12). The deficiency in IL-8 release and most other phenotypical deficits of the HP0858 insertion mutant were restored to a close to parental phenotype by complementation, expressing *H*. *pylori* HP0858 under the control of the *cagM* promoter, which was shuttled into the *rdxA* locus of strain N6 by allelic exchange mutagenesis (Fig 1C and 1D; S2 and S3 Figs). *H*. *pylori* mutants deficient in an essential core component of the CagT4SS, CagY (HP0527) (Fig 1D), or a *cag*PAI-deletion mutant (S6I Fig) were not able to trigger IL-8 secretion to a major extent. Adherence of the bacteria to gastric epithelial AGS cells was not abolished by the mutations (Fig 1G; S2H Fig). In particular for the HP0858 mutant, bacterial adherence was not reduced in comparison to the parent. In the Lipid A-responsive MD2- and TLR4-positive human monocyte/macrophage cell line THP-1 [65], cell activation upon coincubation with live bacteria was also higher for *H*. *pylori* with an intact HP0858 gene and a functional *cag*PAI (S4 Fig) in contrast to the HP0858 or *cagY* mutants. As shown for *Neisseria meningitidis*, some bacterial species are able to secrete activating LPS metabolites into the growth medium [66]. However, adding sterile-filtered culture supernatants of *H*. *pylori* strains or core heptose mutants to AGS cell medium or transfecting them did not lead to significant IL-8 release or NF-κB induction (S5 Fig).

We generated enzymatically treated lysate preparations (ETL; Methods) from *H*. *pylori* which are enriched in LPS components and depleted in proteins, RNA and DNA. Separating these preparations on glycan-detecting silver-stained SDS gels revealed that band patterns corresponding to rough (core) LPS were still detectable in the HP0858, HP0857, HP0860, and HP0859 mutants (at a molecular mass of < 6 kDa), while the biosynthesis of LPS outer chains appeared to be impeded (Fig 1F). In the lysates prepared from the HP0858-complemented strain, the phenotype of outer chain LPS as detected in the silver-stained band patterns remained altered in comparison to the parent (Fig 1F).

**Fig 2:**
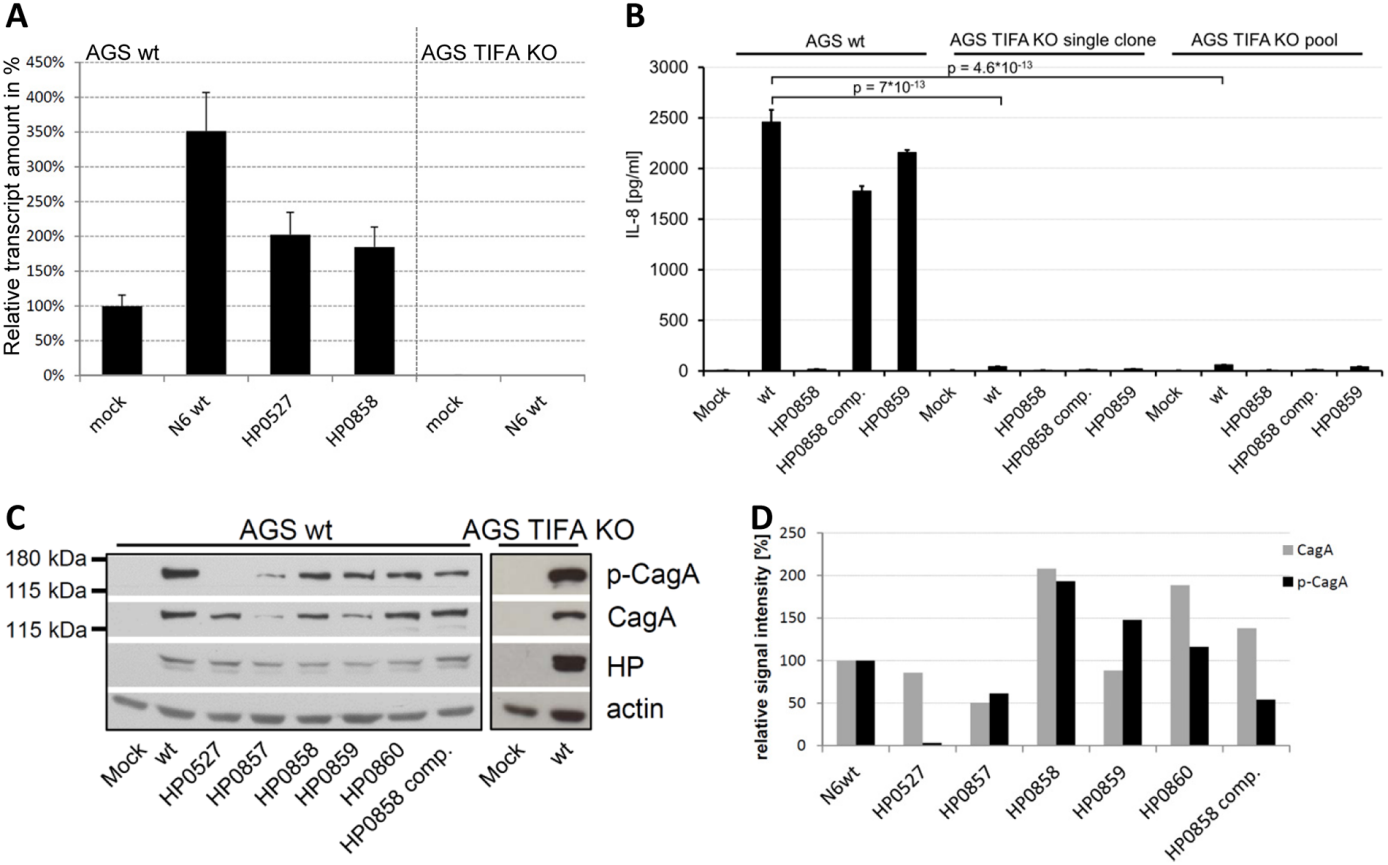
*H*. *pylori* IL-8 activation is abolished in AGS TIFA k/o cells, while *H*. *pylori* CagA translocation is independent of TIFA expression, IL-8 induction, and core heptose biosynthesis pathway intermediates. **A)** qRT-PCR detection of TIFA transcript in AGS wt and AGS TIFA k/o (KO) cells. Cells were mock-incubated or cocultured for 2 h with *H*. *pylori* strain N6 wild type (wt), isogenic *cagY* mutant (HP0527) and heptose pathway *hldE* (HP0858) or *rfaD* (HP0859) mutants as indicated. The TIFA transcript amounts are given in % of the mock-coincubated AGS parental cell, which was set to 100%. **B)** IL-8 induction in AGS CRISPR-Cas9 TIFA knock-out (KO) cells. Indicated *H*. *pylori* strains (N6 wt and isogenic core heptose mutants) were coincubated for 4 h at an MOI of 25 bacteria per cell with AGS parental cells, TIFA k/o cell single clone and TIFA k/o cell pool. HP0858 comp. is the complemented strain. Significance of differences (p) between N6 wt coincubated wild type and k/o cells were calculated by Student‘s *t*-test. **C)** CagA translocation by *H*. *pylori* N6 and isogenic *cagY* (HP0527; CagT4SS functional negative control) and LPS core heptose biosynthesis mutants in AGS cells and AGS TIFA KO cells. AGS cells were coincubated with *H*. *pylori* strain N6 wt or isogenic mutant bacteria for 4 h. 20 μg soluble protein (cleared cell lysates) per lane was separated on an SDS gel and blotted to nitrocellulose membrane. Western blots were incubated with antibodies as indicated. HP signifies the antiserum detection of heat-stable *H*. *pylori* surface antigens (Dako Cytomation, for antibodies see S6 Table) and was used as a universal control for amounts of invariable *H*. *pylori* proteins in the preparations. Actin detection was used as a loading control for amounts of AGS cell proteins. **D**) densitometric quantitation of CagA and p-CagA (CagA translocation) of Western blot results shown in panel **C** (see Methods). The intensity values were normalized to human actin and to HP invariable protein for each condition and are depicted in % of the positive control (AGS cells coincubated with *H*. *pylori* N6 wild type bacteria), which was set to 100%.

**Fig 3:**
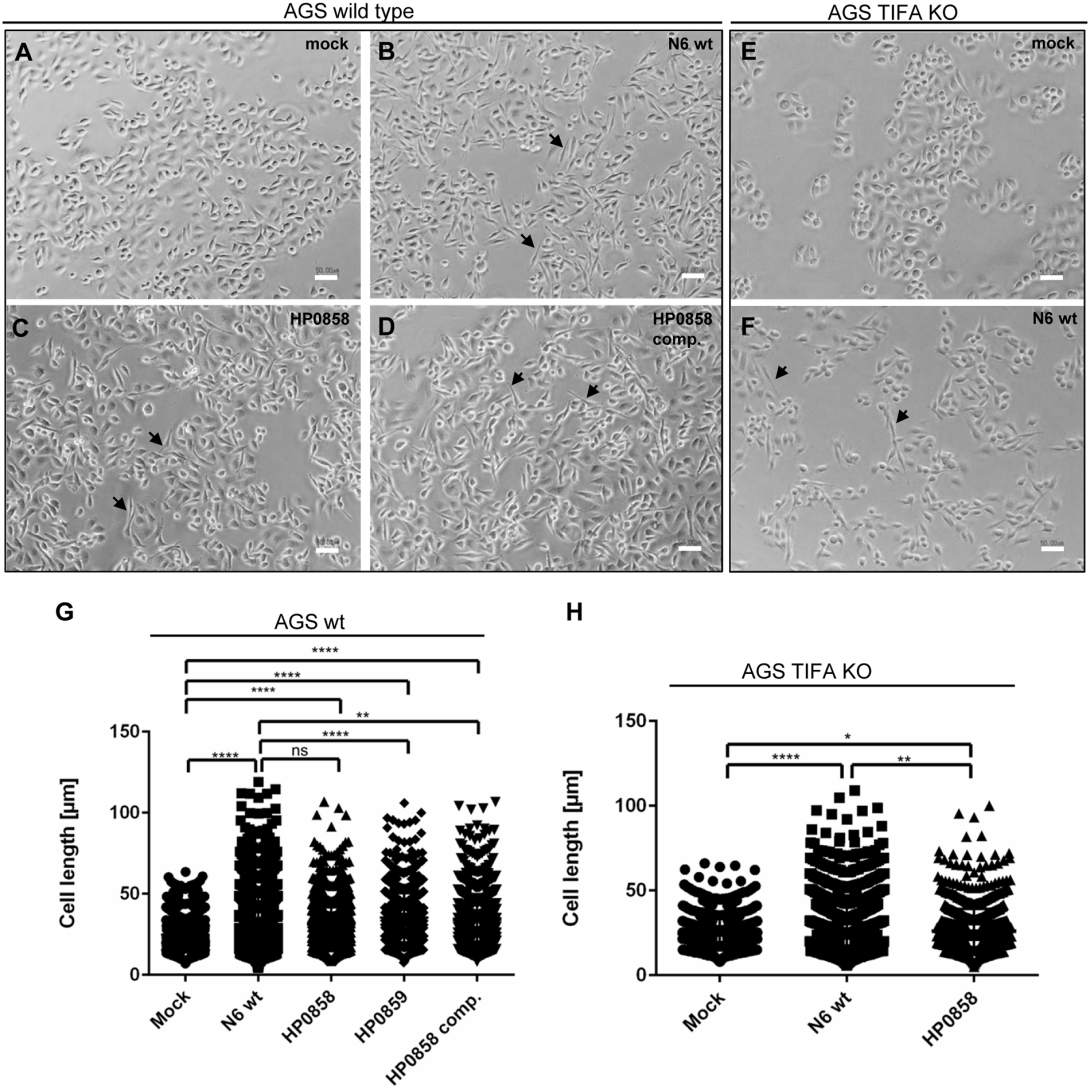
*H*. *pylori* core heptose mutants are able to induce the hummingbird phenotype in human gastric AGS cells (wild type and CRISPR/Cas9 TIFA k/o) independently of IL-8 and TIFA. **A**) mock-coincubated AGS wild type (wt) cells; **B**) AGS wt cells coincubated with strain N6 wt bacteria; **C**) AGS wt cells coincubated with strain N6 HP0858 (*hldE*) mutant bacteria; **D**) AGS wt cells coincubated with N6 HP0858 complemented strain; **E**) mock-coincubated AGS TIFA CRISPR/Cas9 k/o cells; **F**) AGS TIFA k/o cells coincubated with N6 wt. Bacteria were coincubated with the cells for 4 h after centrifugation, fixed and microscopic images acquired. Black arrowheads designate some hummingbird phenotype cells for clarity. Size bars (white) represent 50 μm. **G**), **H**) Quantitation of hummingbird phenotype in bacteria-coincubated AGS wt cells (**G**) or AGS TIFA k/o cells (**H**) as shown in panels **A**) through **F**). 1,000 cells for each condition were quantitated for cell length using ImageJ (as detailed in Methods). Mutants (of strain N6) coincubated with the cells shown in panels G) and H) are indicated by the respective gene numbers. Statistically significant differences between mock-coincubated and bacteria-coincubated cells are shown above the graphs (*p<0.05, **p<0.01, **** p< 0.001, calculated by non-parametric Kruskal-Wallis test; ns = non-significant).

Effector protein CagA translocation into AGS cells, which is another functionality of the CagT4SS, was intact in the HP0857 and HP0858 insertion mutants, despite their loss of the IL-8 secretion phenotype (Fig 2C and 2D). If phospho-CagA as a measure of translocated CagA was normalized to both loading control (actin) and invariable *H*. *pylori* protein bands, CagA translocation was reduced in the HP0857 mutant but not in the HP0858 mutant (densitometry results in Fig 2D). In agreement with this, wild type strains and HP0858 mutants significantly induced the CagA-dependent hummingbird phenotype in AGS cells (Fig 3A to 3G; S6A–S6G Fig). AGS cells coincubated with CagA-deficient or *cag*PAI-deleted mutants (*H*. *pylori* strain 88–3887) showed the expected significant loss of hummingbird phenotype (S6H Fig).

### Host adaptor protein TIFA is crucial for the induction of IL-8 secretion by *H*. *pylori* in human epithelial cells, but not for CagA translocation

**Fig 4:**
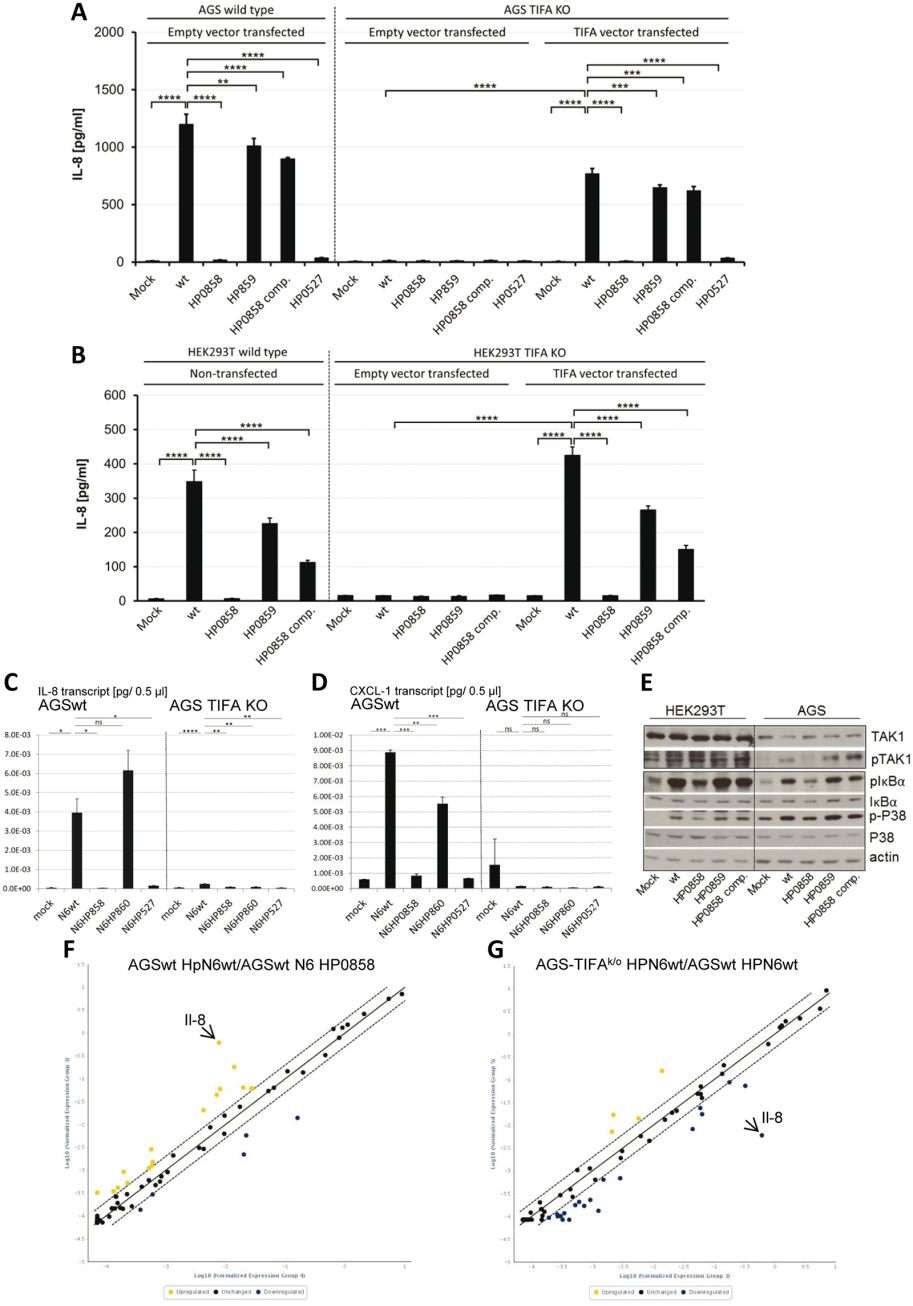
Results of coincubation of parental, TIFA k/o and TIFA-complemented cell lines with *H*. *pylori* and its core heptose LPS biosynthesis mutants and detection of downstream signaling. **A)** AGS **B)** HEK293T parental and CRISPR-Cas9 TIFA k/o cells (pool) were transiently transfected with either an empty vector or a vector expressing human TIFA, by lipofectamine 2000 (HEK) or nucleofection (AGS). On the next day, parental, TIFA k/o and TIFA-complemented cells were coincubated with *H*. *pylori* of indicated genotypes (mutants indicated by respective gene numbers) at an MOI of 25 for 4 h. Cell supernatants were analyzed for IL-8 secretion by ELISA. Statistical significance of differences was determined using two-tailed, non-paired Student's *t*-test (**p<0.01, ***p<0.001, ****p<0.0001). Panels**C)** and **D)** show qRT-PCR data, for il-8 **(C)** and cxcl-1 **(D)** transcript amounts for a panel of bacteria-coincubated AGS parental (wt) and TIFA k/o cells (*H*. *pylori* strain N6 and isogenic mutants). HP0527 = *cagY* inactivation mutant of strain N6, which was used as a control condition of a *cag*PAI functional-negative mutant in cell coincubations. Panel **E)** shows a Western blot to detect downstream signaling in AGS and HEK293T cells upon *H*. *pylori* coculture, dependent on HP0858 (HldE) activity. Cells were coincubated with *H*. *pylori* N6 and isogenic mutants for 4 h. Equal amounts of cellular proteins (20 μg) were separated on SDS gels, blotted and probed with antibodies as indicated to detect the *cag*PAI-dependent downstream activation of p38, TAK1 and IκBa (as reported in [50]). Actin was detected as a loading control. LPS core heptose biosynthesis mutants in all panels are designated with gene names according to the nomenclature of strain 26695 as outlined in Fig 1. **F), G)** results of RT^2^ Profiler RT-PCR arrays in 96 well format (Innate and Adaptive Immune Responses transcript panel selection; Qiagen.com). PCR arrays were performed on cDNA preparations of AGS cells (parental and TIFA knock-out (KO), coincubated with *H*. *pylori* N6 and its isogenic HP0858 mutant. Yellow dots represent over-expressed genes for condition on y-axis. Blue dots represent under-expressed genes for condition on y-axis. Panel **F)** shows the pairwise comparison of amounts of arrayed transcripts between AGS parental cells, coincubated with *H*. *pylori* N6 wild type (wt), versus the same cells coincubated with N6 HP0858 mutant. Panel **G)** depicts the pairwise comparison of transcript amounts between AGS parental cells coincubated with N6 wt and AGS TIFA k/o cells coincubated with the same strain (see also S8 Fig for extended results; full results are summarized in S2 Table).

A recent report described that HBP of various Gram-negative bacteria induces cellular signaling involving the human cytoplasmic adaptor TRAF-interacting protein with an FHA domain (hTIFA; [66]). In order to verify whether this adaptor protein also plays a role in *H*. *pylori cag*PAI-dependent signaling and IL-8 release, we generated genetic knock-out lines of TIFA by CRISPR/Cas9 methodology in two human epithelial cell lines, HEK293T and AGS. CRISPR-Cas9-mediated insertional inactivation mutagenesis of hTIFA (Methods) generated homozygous TIFA-deficient cells, which did not produce TIFA transcript in contrast to the parental cells (Fig 2A). The k/o cells had no major transcript deficiencies or transcript losses in a panel of tested receptor and adaptor protein genes and downstream target genes of the innate and adaptive immune responses (see below). Inactivation of TIFA almost completely abolished the release of IL-8 from AGS and HEK293T cells in response to wild type *H*. *pylori* (Figs 2B, 4A and 4B). This result was demonstrated both with single cell TIFA k/o clones and with a knock-out cell pool (Fig 2B), in which the outcome of off-target effects introduced by the CRISPR/Cas9 approach should be minimized. In TIFA k/o cells, similarly to the parental bacteria, LPS heptose core mutants in either HP0860 or HP0859 (both active for IL-8 induction in wild type cells), did not induce IL-8 (Fig 4A and 4B).

Interestingly, wild type, *cag*PAI-positive *H*. *pylori* translocated CagA regardless of the presence of TIFA (Fig 2C and 2D) and produced a strong CagA-dependent hummingbird phenotype (Fig 3H). In our hands, the core heptose mutants, including the HP0858 mutant and HP0858-complemented strain, also significantly displayed the hummingbird phenotype both in wild type and TIFA k/o cells (Fig 3C, 3D, 3G and 3H). Engineered TIFA deficiency in our CRISPR-Cas9-mutated AGS (Fig 4A) and HEK293T cells (Fig 4B) was complemented back to a functional wild type phenotype by transient transfection of an hTIFA expression plasmid. IL-8 secretion induced by HP0858 and *cag*PAI-positive *H*. *pylori* wild type strains was thereby fully restored in the complemented HEK293T cells (Fig 4B) and almost fully in the hTIFA-complemented AGS cells (Fig 4A), which are more challenging to transfect efficiently. TIFA-mutated AGS cells showed detectable expression of TIFA when complemented with hTIFA, while native TIFA was not detectable by the available commercial antibodies (S7 Fig).

### Characterization of the *H*. *pylori cag*PAI-mediated cellular signaling that is dependent on active LPS core biosynthesis genes and TIFA

The signaling pathways induced by *H*. *pylori* in gastric epithelial cells governed by the *cag*PAI are partially known. Therefore, we verified whether *H*. *pylori* parental strains and isogenic inner core heptose biosynthesis mutants were able to switch on early innate signaling events that had previously been reported to occur downstream of cellular activation by the *cag*PAI, such as NF-κB signaling [34;51;67] and TAK1 and Map kinase phosphorylation [50]. We tested phosphorylation of p38 and TAK1 kinases, and IκBa phosphorylation upstream of NF-κB activation in Western blots as markers for upstream signaling. In addition, transcript analyses of downstream activated human genes (e.g. il-8) were performed by qRT-PCR for both wild type AGS and TIFA k/o cells (Fig 4C and 4D).

*cag*PAI-dependent cell activation of TAK1 and p38 kinases (phosphorylation) as well as IκBα phosphorylation were present in AGS cells coincubated with *H*. *pylori* wild type bacteria for 4 h, but were reduced or even abolished (TAK1) in cells cocultured with the isogenic HP0858 mutant (Fig 4E). In contrast, the HP0859 mutant, functionally downstream of *hldE* in the heptose-bisphosphate biosynthesis pathway, as well as the HP0858-complemented strain exhibited no deficiency in kinase phosphorylation and IκBα activation (Fig 4E). In HEK293T cells, P-p38 and P-IκBα were clearly HP0858-dependent as well, while TAK1 activation was less conclusive for the different *H*. *pylori* strains at 4 h coincubation (Fig 4E).

With regard to characterizing the TIFA-dependent nature of the signaling, we compared selected transcript amounts between AGS wild type and TIFA k/o cells by 96-well qPCR arrays (Qiagen RT^2^ Arrays; Methods), in order to verify whether the k/o mutation caused substantial differences in transcript amounts in the absence or presence of bacteria. We found some transcript differences between wild type and k/o cells, in particular between wild type *H*. *pylori*-coincubated AGS wild type and wild type *H*. *pylori*-coincubated AGS TIFA k/o cells (Fig 4C, 4D, 4F and 4G; S8 Fig; S2 Table). However, no transcript losses except for TIFA (Fig 2A) were detected (for full array results see S2 Table), suggesting that the inactivation of TIFA might be directly causal for the altered phenotype upon *H*. *pylori* coculture. Arrays and selected qPCRs revealed TIFA-dependent differences of the cellular transcript response upon activation by wild type *H*. *pylori* (Fig 4; S8 Fig; S2 Table). We also performed qPCR quantifications of selected transcripts in cells cocultured with live *H*. *pylori* or mock-coincubated cells. Transcript amounts of IL-8 and CXCL1 (Fig 4C and 4D) were most significantly different between mock-incubated wild type AGS cells, *H*. *pylori*-coincubated wild type AGS, and *H*. *pylori* coincubated TIFA k/o AGS cells in our panel of quantitatively tested genes (IL-1β, IL-8, CXCL1, NOD1, HLA-A, C3, IFNAR1, IFNA1, TLR9, IL-13). Coincubation of wild type AGS cells with *H*. *pylori* HP0858 mutants also confirmed the loss of respective downstream transcript activation in comparison to AGS cells cocultured with wild type bacteria (Fig 4C, 4D, 4F and 4G; S8 Fig; S2 Table).

**Fig 5:**
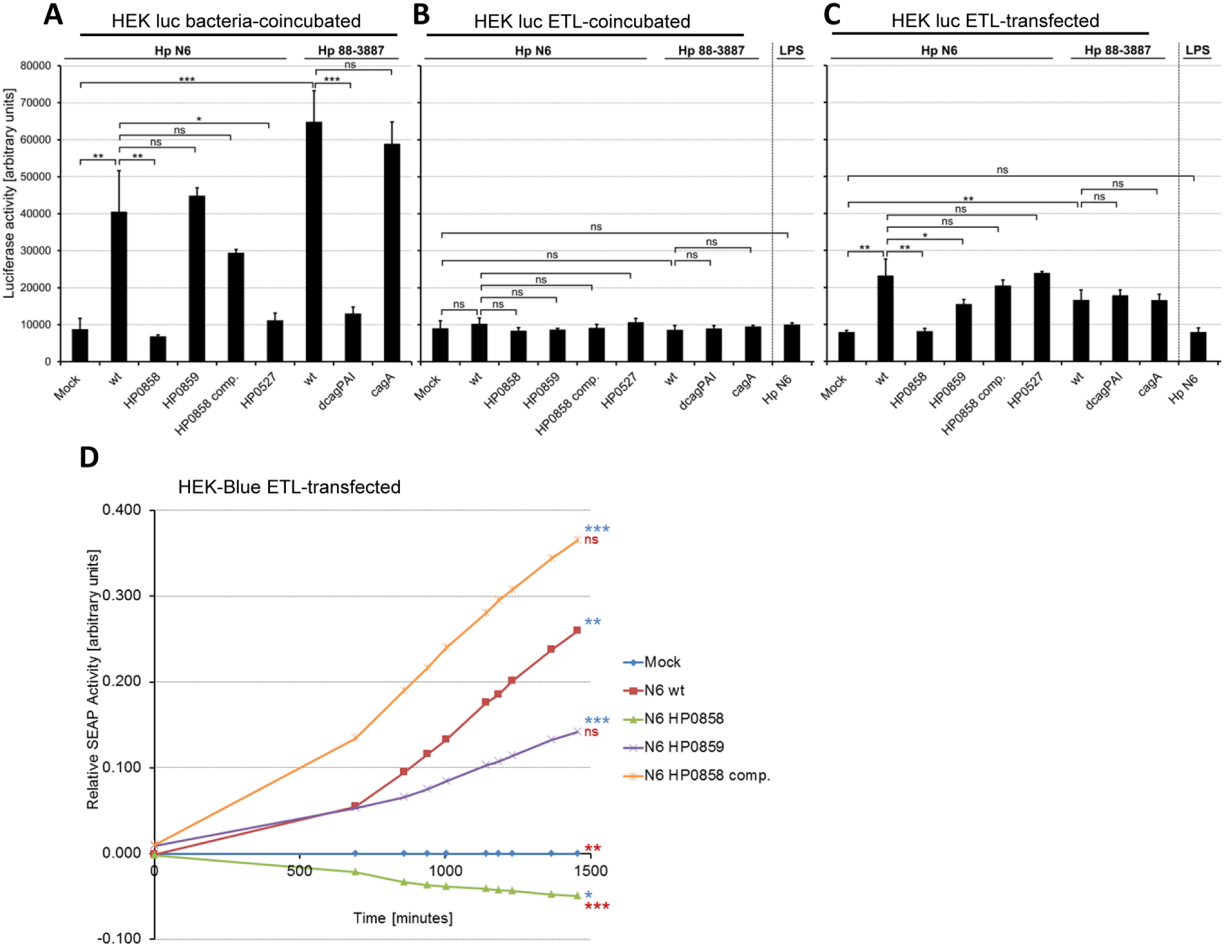
*H*. *pylori* soluble non-proteinaceous metabolite of the HldE-dependent heptose phosphate reaction activates cells when experimentally transfected. **A), B), C)** HEK293T luciferase reporter cell transfection with treated lysate ETL preparations of *H*. *pylori* strains reveal HldE-dependent activation in a transient transfection setting using NF-κB luciferase plasmid (pNFκB-luc). **A**) transiently-transfected HEK293T luciferase reporter cells were coincubated for 3 h with live *H*. *pylori* of either wild type strain N6, its isogenic heptose core pathway mutants or the *cag*PAI function-negative mutant HP0527, as indicated, or parental strain 88–3887, its isogenic *cag*PAI deletion mutant (dcagPAI), or *cagA* mutant, respectively. **B**) transiently transfected HEK293T luciferase reporter cells were coincubated with ETL preparations of *H*. *pylori* strains, heptose pathway mutants, or *cag*PAI mutants added to the cell medium in the absence of transfection agent for 3 h (see silver-stained gel of ETL preparations in Fig 1); **C**) in the third assay set-up, transiently transfected HEK293T cells were super-transfected with ETL preparations of *H*. *pylori* strains and their isogenic mutants. In B) and C, ultrapure *H*. *pylori* N6 wt LPS was also coincubated or transfected as an additional control. Cells in **A**), **B**), **C**) were incubated prior to the luciferase measurement for 3 h. All assay conditions were measured in triplicates. Two-tailed, non-paired Student’s *t*-test indicates significant differences of *p<0.05, **p<0.01, ***p<0.001, ns = not significant. **D**) time-dependent activation of HEK-Blue Null1 SEAP reporter cells (Invivogen) after transfection with treated lysate preparations (ETL) from *H*. *pylori* N6 and its isogenic core heptose biosynthesis mutants. Cell activation by release of secreted alkaline phosphatase into the medium was monitored over a time course of 25 hours post transfection using HEK-Blue real time detection medium (Methods). All conditions were assayed in triplicates. Statistical significance of differences in D) was calculated by Student’s *t*-test for comparisons between mock and all other conditions (blue symbols) and HPN6 wt and all other conditions (red symbols); ns = non-significant; **p<0.01; ***p<0.001; ****p<0.0001.

In addition, we tested whether enzymatically treated lysate (ETL) preparations from *H*. *pylori* strains and isogenic inner core heptose biosynthesis mutants (Methods) were able to induce NF-κB-dependent signaling events similar to the *cag*PAI-dependent responses in host cells in contact with live bacteria. We used two different NF-κB reporter systems, HEK293T cells transiently transfected with NF-κB luciferase reporter plasmid, or HEK-Blue Null1 cells, which possess a stably integrated secreted embryonic alkaline phosphatase (SEAP) gene as an NF-κB- and AP-1-dependent reporter system. Live bacteria activated both reporter cell systems in a *cag*PAI- and HP0858-dependent manner (Fig 5A; S9B Fig), reflecting their potential to activate various cells as shown above. Adding ETL (Fig 1F), which were extracted from the same strains and mutants, including ETLs from a functionally T4SS-deficient mutant (*cagY*/HP0527) or a *cag*PAI deletion mutant, into cell medium in the absence of transfection agent did not reveal a significant NF-κB activation for any strain’s lysate (Fig 5B). In contrast, transfecting ETLs produced from *H*. *pylori* wild type bacteria, *cag*PAI deletion mutant as well as from a *cagA* mutant significantly activated NF-κB reporter cells, while transfecting ETL from a HP0858 mutant did not activate (Fig 5C and 5D). Ultrapure LPS prepared from the N6 parental strain, which contains enriched LPS chains but lacks small metabolites due to multiple extensive dialysis steps, did not activate cells upon transfection in the same setting (Fig 5C). NF-κB-mediated luciferase activation by transfected *H*. *pylori* wild type ETL was concentration-dependent, while transfected ETL from HP0858 mutants did not activate cells significantly at any transfected concentration (S9A Fig). Transfecting of ETL from *H*. *pylori* inner core heptose biosynthesis pathway mutants activated reporter cells to different extents: while transfected HP0858 mutant ETL did not activate, transfected HP0859 mutant ETL significantly activated cells, similar to transfected ETL from wild type or a *cag*PAI deletion mutant (Fig 5C; S9C Fig). ETL from a HP0858-complemented strain activated at the same level as wild type ETL (Fig 5C and 5D). We also assessed various different *H*. *pylori* wild type strains for differences in cell activation potential by live bacteria in comparison to transfection of their respective ETL preparations in HEK-Blue reporter cells. The result of this experiment revealed that the differential activation potential by the live bacterial strains did not correspond to the activation elicited by transfection of their purified ETLs (S9D and S9E Fig). Transfected ETLs from all wild type isolates were able to induce NF-κB or AP-1 activation in comparison to mock-transfected cells, but to very different extents (S9D and S9E Fig).

## Discussion

*H*. *pylori* LPS biosynthesis and strain-specific variations in LPS phenotype have been well characterized [15;16;68–71]. While the fundamental biosynthetic pathways of *H*. *pylori* lipid A and LPS core are quite conserved as in other gram-negative bacteria [16;59;72–74], O-chain LPS modifications in *H*. *pylori* can be mediated by unusual enzymes and are very strain-variable [16;54;61;75–77]. O-side-chain modifications of *H*. *pylori* LPS can mimic human glycans and may also contribute to dampening innate immune responses, possibly via lectin signaling [78]. While *H*. *pylori* purified LPS or lipid A possess a much-reduced capacity to induce proinflammatory signaling by Toll-like receptors (TLR4 or possibly TLR2) when applied to cells from the outside [14;78–81], the *cag*PAI elicits strong cell-activating response pathways by a combination of mechanisms [23;24;33;47;82], including early, mainly CagA-independent signaling [33] and later, CagA-dependent or -independent enhancement [32,49;83;84]. Previous studies proposed that the strain-variable phosphorylation status of *H*. *pylori* LPS contributes to the strength of the signal during *H*. *pylori*-host interaction [52;53;85]. Those studies together with recent findings that the heptose biosynthesis metabolite HBP may contribute to intracellular host cell activation by Gram-negative bacteria [58;66], prompted us to test the hypothesis that *H*. *pylori* LPS inner core heptose biosynthesis might be involved in early host cell activation via the CagT4SS. We report here that one of the major, so far unknown active *H*. *pylori*- and *cag*PAI-mediated cell activation pathways driving NF-κB potentiation and IL-8 secretion is due to LPS inner core heptose biosynthesis, likely through the transport of the heptose intermediate metabolite HBP into host cells via the CagT4SS.

*H*. *pylori* bacteria deficient in the bifunctional LPS biosynthesis enzyme *hldE* (*rfaE*, gene HP0858 in strain 26695 [86]), which abolishes the biosynthesis of HBP, would, by inference from the LPS biosynthesis pathways of other Gram-negative bacteria [87], accumulate the intermediate metabolite sedoheptulose-7-phosphate. Such *H*. *pylori* HP0858 mutants were no longer able to activate the transcription factors NF-κB (and AP-1) and could not elicit the release of IL-8 in human (gastric) epithelial cell lines. The HP0858 mutants obtained in two strains, N6 and P12, produce rough instead of smooth LPS; the bacteria are viable, but grow markedly slower and are less resistant to antibiotic exposure than the parental strains. We did not succeed to generate *hldE* disruption mutants by allelic exchange in various other, highly DNA-uptake competent, *H*. *pylori* isolates, unless a second copy of the gene was provided in a different genetic location. This also underlines a high sensitivity of *H*. *pylori* inner heptose core mutants to environmental stress conditions, even leading to an in-vitro-lethal phenotype in a strain-specific manner. Impaired stress susceptibility phenotypes were previously reported for inactivation mutants in two other *H*. *pylori* genes in the same biosynthesis pathway, *gmhA* (HP0857; [59]) and *rfaD* (HP0859; [60]). These results suggest that *hldE* may be essential in vitro in some *H*. *pylori* strains. However, this study and a systematic insertion mutant analysis by Salama and colleagues confirm that mutants in the gene can be viable [56]. Prior studies have even proposed that *H*. *pylori* mutants in the pathway are generally lethal, because attempts failed to inactivate a proposed *rfaC*/*waaC*-homologous gene (HP0279), encoding a presumed heptose transferase in strain 26695 [61;72]. Mutants which harbor deficiencies in L,D-heptose biosynthesis genes in other bacterial species can be viable in vitro [66;88], although some species appeared not to tolerate the loss of LPS inner core L,D-heptoses, such as for instance in a *Pseudomonas aeruginosa waaP* mutant [89]. Genomic analyses of the heptose biosynthesis clusters did not reveal a clear-cut cause for strain-specific differences between *H*. *pylori* isolates to tolerate gene inactivation in the L,D-heptose pathway. The *H*. *pylori* core heptose coding gene cluster harbors quite a high number of coding and non-coding nucleotide polymorphisms between strains, higher than observed before for proposed common housekeeping genes [8]. Within the cluster, the HP0858 coding regions were the most strain-variable sequences, including multiple non-synonymous polymorphisms. Substantial amino acid variation between HP0858 protein sequences suggests strain-specific differences in the functionality of the protein. Since the coding genes of the cluster are closely adjacent to each other or even partially overlapping, without intergenic spacer segments which might contain regulatory elements, no prediction can currently be made about polymorphisms that may influence strain-specific transcriptional regulation. Reporter cells transfected with equivalent amounts of treated lysates from natural *H*. *pylori* HP0858-variant wild type isolates showed quantitatively different responses, which did not correlate with the activating cell response towards coincubations with the respective live bacteria. This surprising finding suggests to us that the fine-tuning of cell activation by live *H*. *pylori* can be achieved by a combination of heptose pathway activity and the modulation of CagT4SS transport activity.

In our study, an insertion mutation in the last gene of the biosynthesis cluster, HP0857 (functionally the first gene in the pathway), like the HP0858 mutant, abolished the *cag*PAI-dependent IL-8 secretion phenotype. In contrast, a mutant in the first upstream gene of the cluster, HP0860, whose gene product catabolizes the dephosphorylation of heptose-1,7-bisphosphate to heptose-1-phosphate, retained the potential to significantly induce IL-8 secretion and NF-κB activation, although it exhibited a slow-growing, LPS-rough phenotype as the HP0858 mutant. The N6 HP0859 mutant, generated in our laboratory and by others [60], showed here similar cell activation phenotypes as the HP0860 mutant, almost at the same level as the parental strain. The two latter pathway mutants, although probably not able to form a complete heptose LPS core and O-chain LPS, since subsequent steps of the heptose biosynthesis are interrupted, would not abolish the formation of the metabolic intermediate heptose-1,7-bisphosphate. The previous characterization of *H*. *pylori* 26695 HP0859 or HP0857 insertion mutants reported stunted growth and reduced *H*. *pylori* adherence to AGS cells to one tenth or 50% of the parental strain, respectively [59;60]. While we also observed a strong growth attenuation of the mutant in HP0858 and other mutants in the pathway, in particular in liquid culture, we noted no significant impairment in cell adherence of the pathway mutants HP0858 or HP0859, which corroborated previous adherence data on various *H*. *pylori* LPS O-chain mutants [90]. The differences reported between various studies in the effect of core heptose mutagenesis on bacterial adherence might be due to strain-specific phenotypes, possibly by interference with outer membrane integrity, membrane protein insertion, or to differential effects of growth conditions. Our N6 HP0858-complemented strain recovered the ability to activate cell responses almost to the level of the parental strain (in both AGS and HEK cells), although it did not recover a wild type-like LPS phenotype, presumably by an imbalanced expression of the gene from the heterologous *rdxA* locus.

Interestingly, in our study, all mutants and the complemented strain retained the ability to translocate CagA into human gastric epithelial cells. The CagA translocation ability was not markedly reduced for the HP0858 mutant in comparison to wild type bacteria under our test settings. In tune with this and differently from what was observed by others for the HP0859 and HP0857 mutants [59;60], both the N6 HP0858 and HP0859 mutants were still able to significantly elicit the hummingbird phenotype in the target AGS cells, parental and TIFA k/o cells, after four hours of coincubation at an MOI of 25. Strain-specific growth defects, differences in experimental settings and the less pronounced hummingbird phenotype might be potential reasons why divergent phenotypes were reported in previous publications. The hummingbird phenotype induced by the HP0858 heptose core mutant was slightly less pronounced (non-significantly different) than for the wild type bacteria in AGS wild type cells, however significantly reduced but still present in TIFA cells. We cannot exclude that this effect was due to TIFA deficiency, however we suggest that the differences are due to experiment-specific and pleiotropic effects introduced by fitness defects of the mutant. In confirmation of this hypothesis and previous reports [59;60;62], we determined increased antibiotic sensitivities of the inner core heptose mutants. Alternatively, although we did not detect it for the HP0858 mutant, we cannot exclude a slight decrease or qualitative difference in cellular translocation activity of the CagT4SS [90;91] by inner core heptose deficiency.

We also demonstrated here using CRISPR/Cas9 inactivation and subsequent reconstitution by complementation that TIFA, an important forkhead domain signaling adaptor connected to TRAF6 and inflammasome signaling in eukaryotic cells [66;88;92;93], contributes strongly to IL-8 secretion and NF-κB activation mediated by the *H*. *pylori* CagT4SS. The connected findings that the full range of cell responses to *cag*PAI+ *H*. *pylori*, which require the presence of cellular TIFA and lead to IL-8 induction, were not essential for CagA translocation and the hummingbird phenotype showed convincingly that these processes are separate functional activities of the CagT4SS that can be governed independently from each other.

TIFA is involved in immune activation by TRAF2 and TRAF6 interaction [94–96] and can be phosphorylated at Thr9, which mediates its oligomerization, triggering the activation of NF-κB [92]. These known activation mediators fit well with the already acknowledged essential role of TRAF6 and confirmed downstream factors such as TAK1 and NF-κB [49;50] in this newly described mode of *H*. *pylori*-triggered cell activation, which requires TIFA, HP0858 activity and the *cag*PAI.

Adding soluble components from bacterial culture supernatants or enzymatically treated lysate preparations of *H*. *pylori* parental strain(s) or LPS heptose core mutants to cells from the outside was not able to induce TIFA-dependent IL-8 secretion, neither in AGS or HEK cells, nor cell activation in NF-κB reporter cell lines. In contrast, transfecting treated lysates from both, wild type bacteria or *cag*PAI deletion mutants, activated cells, whereas transfected lysates from HP0858 mutants or transfected ultrapure LPS from wild type bacteria were not able to activate significantly. These findings confirmed that the activation pathway under investigation is independent of Lipid A-driven signaling, but clearly dependent on two factors jointly: a functional *cag*PAI and active HP0858. The conclusion that a soluble LPS metabolite may be translocated via the CagT4SS and activate cells from the inside explains one major activating principle of the *cag*PAI and also reconciles the confirmed low *H*. *pylori* LPS activity on TLRs with very early reports that a secreted, stable product of *H*. *pylori* can activate NF-κB in gastric epithelial cells [97]. Very recent reports implied that the activation of cells by *Shigella* and *Salmonella* also involves the HBP-TIFA pathway [88;98]. The authors speculate that metabolite transport inside cells or the cytoplasm for activation may also require the activity of *Salmonella* and *Shigella* secretion systems (supposedly Type 3), which they did not yet confirm experimentally [88]. For *H*. *pylori*, it seems plausible to speculate that in certain endocytic cell types, the metabolite HBP might even be taken up together with the bacteria, with outer membrane vesicles, or with low amounts of released metabolites, and initiate activation of TRAF6 via TIFA from the bacterial environment. Interestingly, Gaudet and colleagues [98] very recently identified a sequential activation hierarchy between TIFA and NOD1 in the target cell. Since NOD1-dependent NF-κB activation by *H*. *pylori* via the CagT4SS has been described before [46;99], this possible activation link should also be investigated in gastric epithelial cells, although we currently did not find evidence for significantly changed nod1 transcript in TIFA k/o cells.

Since the inactivation mutagenesis of HBP biosynthesis pathway genes is difficult in many strains and the obtained rare mutants are generally slow-growing and stress-sensitive as described by us and others, we argue that it will unfortunately not be very informative to use these few strain-specific mutants in animal models of chronic *H*. *pylori* infection. However, testing an HP0858-overexpressing *H*. *pylori* strain in an animal model of the acute infection phase might be feasible and will be worthwhile to pursue in a future study. In conclusion, we demonstrate that IL-8 induction by *H*. *pylori* is almost entirely dependent upon an intact core heptose biosynthesis pathway able to generate HBP. IL-8 induction is likewise almost completely dependent upon cellular TIFA and on a functional CagT4SS. The data strongly suggest that HBP is delivered to the host cell by means of the T4SS, where it is sensed either by TIFA itself, or, more likely, by a yet unknown intracellular sensor for this novel MAMP. Our present results open up a novel field of study to dissect the manipulation of the TIFA-dependent cell activation pathways by *H*. *pylori* and their potential role in chronic inflammation, bacterial persistence and carcinogenesis.

## Materials and methods

### Bacterial strains and culture conditions

*H*. *pylori* wild-type strains N6 [63], 88–3887 (26695A [8]) and P12 [64] were used for genetic manipulations, protein analysis and eukaryotic cell infections. The two strains harbour a functional *cag* pathogenicity island (*cag*PAI) in their genome, encoding the Cag T4SS [8;25] *H*. *pylori* strains and mutants used in this study are listed in S3 Table. Bacteria were regularly grown on blood agar plates (Oxoid blood agar base II, Wesel, Germany) supplemented with 10% (v/v) horse blood and the following antibiotics (all purchased from SIGMA): amphotericin B (4 mg/liter), polymyxin B (2,500 U/liter), trimethoprim (5 mg/liter) and vancomycin (10 mg/liter). Plates were continuously incubated in anaerobic jars under microaerobic atmosphere (10% CO_2_, 5% oxygen, 85% nitrogen) generated by Anaerocult C sachets (Merck, Darmstadt, Germany). Mutant strains were selected and cultivated on 5% horse blood (Oxoid) agar plates mixed with chloramphenicol (10 mg/liter) and/or kanamycin (100 mg/liter or 25 mg/liter). E-tests (using MIC test strips, Liofilchem s.r.l., Roseto degli Abruzzi, Italy) of *H*. *pylori* strains and mutants were performed for tetracyclin and rifampicin on commercial Columbia 5% sheep blood agar plates (Oxoid) without any further addition of antibiotics in the plates. Liquid cultures of *H*. *pylori* strains and mutants were conducted in Brain Heart Infusion (BHI) broth (Oxoid) supplemented with 3% yeast extract (Oxoid) and 5% horse serum (Gibco Life Technologies, Karlsruhe, Germany, # 16050–122) for up to 48 h, starting with an inoculum suspension prepared from 24 h plate-grown bacteria to an initial O.D._600_ of about 0.06. Statistical analysis of differences of growth between strains was determined by two-way ANOVA, followed by a Tukey’s pairwise comparison test (GraphPad Prism) (S1 Table). *E*. *coli* strains DH5α [100] and MC1061 [100] were used for cloning and plasmid propagation. *E*. *coli* strains were propagated on Luria Bertani (LB) plates or in LB broth containing ampicillin (100 mg/liter), chloramphenicol (20 mg/liter) and/or kanamycin (100 mg/liter) as required.

### Standard techniques for DNA and protein analysis

Standard procedures for cloning and DNA analysis were performed according to [100]. DNA modification and restriction enzymes were purchased from Invitrogen (Life Technologies, Germany), New England Biolabs (NEB) or Roche. Isolation of highly pure genomic DNA, purification of plasmid DNA and gel extraction of DNA from enzymatic reactions were accomplished by QIAGEN DNA purification columns (QIAGEN, Hilden, Germany). Plasmids and oligonucleotide primers for cloning, sequencing and site-directed mutagenesis are depicted in S4 and S5 Tables, respectively. PCRs were run in Biometra thermocyclers (Biometra, Goettingen, Germany) using *Taq* polymerase (GE Healthcare). *Pfu* (Agilent Technologies), FastStart HF (Roche) and Q5 (NEB) DNA polymerases were used for accurate amplification of longer DNA fragments and for cloning. Protein separation and detection by denaturing SDS polyacrylamide gel electrophoresis (SDS PAGE) and Western blotting were performed according to standard methods [101;102]. Total protein concentration was measured using the bicinchoninic acid (BCA) assay (ThermoFisher Scientific). Protein samples were separated on 10.6% or 11.5% SDS polyacrylamide gels, depending on the molecular mass of the protein or other molecules to be analyzed. Subsequently, separated proteins were either stained directly, or transferred to nitrocellulose membranes (Schleicher & Schuell, Germany) by tank blotting. Staining was performed using RotiBlue Coomassie Blue staining solution (Roth, Germany). For silver staining, ETL preparations were resolved on 14% SDS gels. The gels were fixed and subjected to a glycan-based (periodic acid) silver staining method modified according to Dubray et al. [103;104]. Antisera and antibodies for immunodetection (S6 Table) were diluted in TBS-T (TBS with 5% skim milk blocker (Biorad) and 0.1% Tween 20 (SIGMA) or TBS-T with Bovine serum albumin (BSA, 5% (SIGMA)). Secondary antibodies were used as conjugates to horseradish peroxidase (HRP). For detection of the HRP signal, membranes were incubated with enhanced chemiluminescent substrate (SuperSignal West Pico, Thermo Scientific) and exposed to chemiluminescence detection films (Amersham Hyperfilm ECL, GE Healthcare). Before reapplication of antibodies, membranes were stripped with Restore Western Blot Stripping Buffer (ThermoFisher Scientific).

### Generation of *H*. *pylori* lysates, enzymatically treated lysates (ETL), culture supernatants and ultrapure LPS

Whole cell lysates were generated from bacteria grown for 1 day on blood agar plates. A bacterial suspension of OD_600_ = 1.7 was generated in Tris/HCl (50 mM, pH 7.5). Bacteria were sonicated twice for two minutes at 4°C (Branson Sonifier 450, Constant Duty Cycle, Output Control 5).

Enzymatically treated lysate (ETL) preparations were generated from bacteria grown for 1 day on blood agar plates as follows. A bacterial suspension of OD_600_ = 2 in PBS was boiled for 10 min, followed by centrifugation at 10.000 x g, 4°C. Supernatant was transferred to a new tube. Protein was digested by 20 μg/mL proteinase K (QIAGEN, Hilden, Germany) at 56°C for 30 minutes. RNA was digested with 11.25 μL/mL of RNaseA (QIAGEN, 10 mg/mL) at 70°C for 15 minutes, and DNA twice with 11.25 μL DNaseI (Roche) at 37°C for 10 minutes. The reaction was again boiled for 5 min and centrifuged for 2 min at 21,000 x g in a table-top centrifuge at room temperature. The supernatant was filtered through a 0.2 μm syringe sterile filter (Millex-GV, SLGV013S) in order to remove any large debris and intact bacterial cells, stored at -20°C and thawed on ice before use. The ETLs should be relatively enriched in non-proteinaceous, non-DNA, non-RNA heat-stable components, e.g. LPS, sugars, lipids and small metabolites. Sterile culture supernatants from bacterial liquid culture were generated after growth to mid-log (24 h) or late log (48 h) phase in BHI medium containing 3% yeast extract and 5% horse serum under microaerophilic conditions. Supernatants were harvested after removing the bacteria by centrifugation and subsequently sterile-filtered through 0.2 μm Millex GV filters (Schleicher and Schuell, Germany). Ultrapure LPS from *H*. *pylori* was generated as previously described, using a modified hot-phenol extraction method [105].

### Construction of insertion and allelic exchange mutants and gene complementation in *H*. *pylori*

HP0857, HP0858, HP0859 and HP0860 mutants were generated by insertion of an *aphaA3* (kanamycin resistance) or *CAT* (chloramphenicol resistance) cassette by allelic exchange mutagenesis in the N6 or P12 *H*. *pylori* strains. Both cassettes are non-polar on surrounding genes if inserted in the orientation of the disrupted gene, since they do not carry a transcriptional terminator [106;107]. For the HP0858, HP0859 and HP0860 mutants, 500 base pair long arms of homology for the gene of interest were amplified from genomic DNA of *H*. *pylori* strain 26695 by PCR with the FastStart High Fidelity polymerase (Roche), introducing restriction sites at the ends of the products. The antibiotic resistance cassettes were either released by restriction digest from a plasmid or amplified by PCR with restriction sites matching the arms of homology. 5´and 3´arms of homology were ligated to the resistance cassette with T4 ligase (Roche). The resulting product was either directly used as a template for PCR or ligated into a plasmid, which then served as a PCR template. For the HP0857 mutant, an expression plasmid containing the HP0857 gene from the *H*. *pylori* 26695 strain was reverse amplified introducing restriction sites in the middle of the gene. The CAT cassette was ligated into the amplified expression plasmid.

PCR products of the region spanning the arms of homology of the gene of interest with integrated antibiotic resistance cassette were introduced and recombined into *H*. *pylori* strains by natural transformation, and desired mutants were selected on blood agar plates with the respective antibiotic. Correct insertion of the resistance cassette in the target gene of the bacterial chromosome was checked by PCR with primers amplifying the whole region and additionally by combining gene-specific and resistance cassette specific primers.

Complementation of HP0858 in *cis* was achieved by integrating an additional copy of the gene into the chromosomal *rdxA* locus, leaving the disrupted gene in the original locus non-functional. The HP0858 gene and the *cagM* promoter from *H*. *pylori* strain 26695 were amplified by PCR with primers introducing a ribosomal binding site and ligated in a plasmid based on pCJ542 [108] in a site that contained a CAT cassette between arms of homology of the *rdxA* gene (pCJ1624). The suicide plasmid was introduced into *H*. *pylori* wild type or isogenic heptose biosynthesis mutant by natural transformation, and mutants having undergone allelic exchange in the chromosomal copy of *rdxA* were selected on blood agar plates containing chloramphenicol or kanamycin and chloramphenicol. The mutants were checked by PCR for correct integration in the *rdxA* site and for retention of the original disrupted chromosomal locus. Primers used for cloning are summarized in S5 Table.

### Coculture of *H*. *pylori* with cells, NF-κB reporter assays and quantification of chemokine secretion

We cocultured *H*. *pylori* strains with different human cell lines: AGS (ATCC CRL-1739); HEK293T (ATCC CRL-2316), or HEK-Blue Null1 cells (Invivogen). We routinely grew the cells in either RPMI 1640 medium (Biochrom, Germany) with 10% fetal bovine serum (FBS, Gibco, Germany # 10270–106) buffered with 25 mM HEPES (Biochrom, Berlin, Germany) (for AGS), or in DMEM (Biochrom, Germany) with 10% FBS (For HEK cells). Cell infection experiments were carried out on subconfluent cell layers (60% to 90% confluence) in 24 or 96 (for reporter assays) well plates. A medium change to fresh medium containing FBS was performed about 60 min prior to the start of each coincubation. Exponentially growing bacteria (after growth on blood plates for 20 to 24 h) were harvested and resuspended in fresh cell culture medium of the respective type (either RPMI 1640 or DMEM), containing 10% FBS, respectively. Cells were cocultured with *H*. *pylori* at different multiplicities of infection (MOI), as indicated in the respective text passages and figure legends. The infection was synchronized by centrifugation of the incubation plates (300 x g, 10 min, room temperature). Cells and bacteria were cocultured for 20 to 25 h (IL-8 secretion and HEK-Blue SEAP detection system), for 4 h (IL-8 secretion and CagA translocation), for 3 h (NF-κB reporter cell assay), or for 2 h (transcript analyses), as indicated in the figure legends. Mock-infected cells (mock) were used as negative controls. Supernatants were harvested, cleared by centrifugation (22,000 x g, 2 min, at room temperature) and stored at -20°C. IL-8 secretion into cell supernatants was determined using a commercial human IL-8 ELISA (BD OptEIA IL-8 enzyme-linked immunosorbent assay kit, BD Pharmingen, San Diego, USA) according to the supplier´s instructions, using appropriate dilutions. IL-8 determination for each sample was performed in triplicates on biological duplicates, and the cell coculture was performed at least two times independently on different days. Student’s *t*-test or ANOVA (GraphPad Prism) were performed for determining statistical significance of cytokine secretion.

NF-κB reporter assays were performed in 96 well plates in triplicates using the HEK-Blue Null1 cells (Invivogen), which express Secreted Alkaline Phosphatase (SEAP) under the control of a minimal interferon-beta promotor containing five NF-κB and AP-1 binding elements. Activation of these cells was quantitated in HEK-Blue Detection reporter medium (Invivogen), for real-time SEAP detection. Colorimetric measurements of the 96 well plates were carried out in a multi-well plate reader (Biotek) at an O.D. of 620 nm at indicated time points. As alternative reporter cells, HEK293T cells transiently transfected with a firefly luciferase reporter plasmid (10 ng of plasmid DNA per well, pNFκB-luc, BD Biosciences) were employed. For the luciferase assay, cells were lysed at 3 h post infection with the Steady-Glo Luciferase Assay System (Promega) according to protocol (Promega). Luciferase readings were recorded in a Victor Wallac 420 multi-well plate reader in luminometer mode.

In order to test the activation potential of intracellularly delivered LPS metabolites, *H*. *pylori* ETL samples were experimentally transfected into HEK NF-κB luciferase or HEK-Blue Null1 SEAP reporter cells using Lipofectamine 2000 transfection agent (Invitrogen). The ETL transfection assays were performed in 96-well plates containing ca. 5x10^4^ cells in 100 μl of culture medium, using 25 μl transfection volumes per well, which included 4 μl of ETL, 2 μl of Lipofectamine 2000 (Invitrogen) and 20 μl OptiMEM medium (Biochrom) without serum. As additional control conditions, sterile-filtered bacterial supernatants from bacteria grown in liquid culture (O.D._600_ = 0.6) were also Lipofectamine 2000-transfected into HEK reporter cells using the same method. Used as a control condition, transfected culture supernatants recovered from N6 wild type bacteria grown in liquid showed only a very weak stimulation potential for NF-κB in comparison to transfected wild type ETL preparations (S5 Fig, see also Results).

### Bacterial cell adherence assay for *H*. *pylori* in 96 well format

To quantitate adherence of *H*. *pylori* bacteria to AGS cells, cells were plated in 96-well plates to ca. 5x10^4^ cells per well (full confluency). Cells were co-cultured with bacteria as described above with an MOI of 25 using bacteria that had been grown on blood agar plates for about 40 to 48 h. Cells with bound bacteria were fixed with a solution of 2% freshly prepared paraformaldehyde (SIGMA) in 100 mM potassium phosphate buffer pH = 7.0 (potassium salts by SIGMA-Aldrich). 50 μL of fixation solution were added per well, followed by one change of fixing agent for two overnight incubations. After fixing, the fixation solution was quenched with 0.1% of glycine prepared in cell culture PBS.

Fixed cells were washed three times with wash buffer (1xPBS, 0.05% Tween-20 (SIGMA) and blocked for 30 minutes with 200 μL blocking buffer (10% FBS in 1xPBS (Biochrom)). After washing four times as before, the cells were incubated for one hour at ambient temperature with 100 μL of anti-*H*. *pylori* antibody (Dako Cytomation, B0471), diluted 1:2,000 in blocking solution. Following four wash steps, the cells were incubated for one hour at RT with 100 μL goat-anti-rabbit horseradish-peroxidase-coupled secondary antibody (Jackson ImmunoResearch), diluted 1:10,000 in blocking buffer. After the incubation, cells were washed seven times for 30 seconds with wash buffer before a final incubation step with 100 μL of BD OptEIA TMB substrate (BD Biosciences) for development. The incubation was stopped after 45 minutes with 50 μL of 1 M H_3_PO_4_ (SIGMA-Aldrich). 140 μL of the reaction was transferred to a round-bottom 96-well plate (Greiner, Germany) and measured at O.D._450_ (reference wavelength 540 nm). As a control for antibody reactivity to the different bacterial strains, bacteria were fixed to gelatin-coated wells of a 96 well plate and subsequently detected by the same method as described above. Statistical significance was determined by Student’s *t*-test.

### *H*. *pylori* CagA translocation assay

Translocation of the CagA effector protein into cells was examined by coculturing *H*. *pylori* strains with AGS cells in 24 well plates. *H*. *pylori* were coincubated with the cells at an MOI of 25 for various coincubation periods as indicated. We removed non-adherent bacteria by washing the cells once with PBS Dulbecco (cell culture grade, pyrogen-free, pH = 7.4, Biochrom, Berlin, Germany). Cells were harvested on ice using lysis buffer (modified RIPA lysis buffer: 50 mM Tris-HCl [pH = 7.5], 150 mM NaCl, 1 mM EDTA, 1 mM EGTA, 0.5% Igepal CA-630 (SIGMA), PhosStop [Roche], CompleteMini protease inhibitor [Roche]) and stored frozen at -20°C. During lysis, cells were placed on ice for 30 min. Cell debris was cleared by centrifugation (22,000 x g, 20 min, 4°C), followed by collecting the supernatants into fresh tubes.

CagA translocation was determined by separating the soluble fraction on 10.6% SDS-PAGE (10 or 20 μg of total protein loaded per lane) and immunoblotting with an affinity-purified anti-phospho-peptide antibody (rabbit anti-*Hp*-pCagA, 1:333; [31]). Signal intensities were validated against detection intensities of total CagA protein on the same blots, using a polyclonal α-CagA antiserum (rabbit α-*Hp*-CagA-antigen, IgG-fraction, Austral Biologicals, San Ramon, USA, 1:10,000). As a bacterial loading control, heat-stable *H*. *pylori* antigens (rabbit α-*H*. *pylori*, DAKO, Denmark, 1:2,500) were detected; as a cellular loading control, we detected human actin (mouse α-actin, Millipore, Schwalbach, Germany, 1:20,000). For the quantification of pCagA protein amounts, the detected signals were scanned to TIFF files and analyzed by densitometry using ImageJ [109]. CagA and phospho-CagA signal intensities were normalized first to loading control (human actin) and then to invariable *H*. *pylori* protein band signal intensities. CagA translocation assays were at least performed twice independently on different days for each tested strain. For references to used antibodies, see also S6 Table.

### CRISPR/Cas9 mutagenesis of human TIFA and complementation by transient plasmid transfection

TIFA knockout cell lines were generated with a CRISPR/Cas9 kit from Origene (KN204357) that disrupts the TIFA gene by replacing the first 104 bases of the coding sequence including the start codon with a GFP-puromycin cassette. AGS and HEK293T cells were transfected with pCas Guide (G1) and Donor vectors (Origene) using Fugene HD (Promega) for AGS cells or Lipofectamine 2000 (Invitrogen) for HEK293T cells. Cells were split over several passages to dilute out transfected plasmid before undergoing selection with 2.5 μg per ml of puromycin (Sigma), until visible colony formation occurred. Single cell colonies were picked with cloning discs (Sigma) and expanded. Pools of remaining puromycin-resistant colonies were also expanded. Integration of donor plasmid DNA into the disrupted TIFA locus was tested with primer combinations producing products either for wild type or integrant loci, enabling discrimination between homozygous and heterozygous gene knockout.

Complementation of TIFA in the knock-out cell lines was achieved by transient transfection of an expression plasmid containing the human TIFA (hTIFA) ORF tagged with Myc-DDK (Origene True ORF Gold, pCMV6_TIFA_Hs, RC204357). Control cells were transfected with an empty expression plasmid (pEF6-V5-empty; [17]). Cells were transfected with 500 ng (AGS) or 100 ng (HEK293T) control or expression plasmid per well in 24-well plates with either Fugene HD (Roche) (for AGS cells), Nucleofector reagents (4D-Nucleofector reagent SF, program DS-135; Lonza) (for TIFA complementation of AGS cells) or Lipofectamine 2000 (HEK293T cells), one day before the coincubation experiments were performed.

### RNA preparation, cDNA synthesis and real-time (quantitative) PCR

Cells were grown in 6-well plates (ca. 2x10^6^ cells per well) under coincubation conditions for 2 h, scraped from the plates and harvested by centrifugation at 22,000 x g for 1 min at 4°C. The cell pellets were snap frozen in liquid nitrogen and stored at -80°C until further use. Total RNA was prepared from each cell pellet using a modified RNeasy spin column protocol (Qiagen, Hilden, Germany) after mechanical homogenization in a Fastprep bead-beater (MP Biomedicals Inc., Santa Ana, CA, USA), at power setting 6 for 45 sec. The amounts and purity of the isolated RNA were determined in a spectrophotometer at 260 nm and on agarose gels. DNA contamination was eliminated by DNaseI treatment with TURBO RNAse-free DNA removal kit (Ambion) according to the manufacturer’s instructions and verified by PCR.

cDNA was synthesized from 1 μg of total RNA, using a combination of random hexamer primers and oligo-dT T12-T18 primers (Invitrogen) and the Superscript III reverse transcriptase (Invitrogen) at 42°C for 2 h.

Quantitative (q) RT-PCR was performed on pretested amounts of cDNA specific for each transcript (between 0.5 and 2.5 μl) according to standard protocols, using gene-specific primer pairs (Qiagen Quantitect primer kit), ultrapure water and SYBR Green Master Mix (Qiagen). qPCR reactions were run and analyzed in a BioRad C1000/CFX96 combined real-time PCR system (BioRad, Hercules, USA). All samples were analyzed as technical triplicates, and standard curves were generated from defined amounts of PCR product of the respective genes to allow quantitation. Values were equalized to 0.5 μl cDNA input and normalized to human GAPDH transcripts of each respective condition, using the hGAPDH transcript quantity of mock-coincubated cells as a reference.

### RT^2^ Profiler quantitative PCR arrays

RT^2^ Profiler PCR Arrays, which contain qPCR primers for selected genes and controls in a 96-well format, were commercially acquired (Qiagen). We used the gene selections of “Innate and Adaptive Immune Responses” (cat.#: PAHS-052Z; details of RT^2^ arrays and selected gene panels see: Qiagen.com; gene list see S2 Table). The Profiler arrays were run using SYBR Green master mix for amplification and detection. Equal amounts of cDNA (0.25 μl), prepared from the different coincubation conditions of AGS wild type and AGS TIFA k/o cells, were amplified in the arrays in a BioRad CFX96 real time PCR machine under standard cycling conditions according to the manufacturer’s instructions. Two qPCR arrays were performed for each coincubation condition, except for the AGS wild type cells, coincubated with *H*. *pylori* N6 wild type bacteria (three arrays). The experimental conditions were ordered into experimental groups (1- control group, additional groups 2 through 5) for the online evaluation (two arrays or three arrays [group 3], respectively, are summarized in each group; for group definitions and input data including numbers of arrays per group, see S2 Table and figure caption to S8 Fig). The evaluation of the RT^2^ results was performed in the online evaluation software RT^2^ Profiler PCR Array Data Analysis v3.5 according to the online instructions at qiagen.com. (Full evaluation results and evaluation details see S2 Table). Three invariable human housekeeping genes (hGAPDH, hHRPT1, hRPLP0) were selected as internal controls for each array and for normalization.

### Transmission electron microscopy

Transmission electron microscopy was performed in a Morgagni TEM 268 microscope (FEI) on samples deposited on formvar-carbon coated copper grids (Plano), negatively stained using phospho-tungstate salt (SIGMA; as a 2% solution in potassium-phosphate buffer, pH = 7.0). Digital images were acquired at a magnification of 8,900-fold.

### Live-dead staining of *H*. *pylori* cells

Bacteria from 24 hour plate cultures were resuspended and diluted in Brain Heart Infusion broth (Oxoid) medium to an OD_600_ = 0.1. 100 μL of the dilution were mixed with 0.5 μL of LIVE/DEAD BacLight Bacterial Viability Kit L7007 (Molecular Probes, ThermoFisher Scientific, USA) and incubated for 30 min. Bacterial smears were prepared and observed in the fluorescence microscope (Olympus IX-40) at a lens magnification of 40-fold, and digital color images were acquired using AnalySIS software (Olympus).

### Quantification of hummingbird phenotype in *H*. *pylori*-infected AGS cells

AGS and TIFA k/o mock and bacterial infected cells were fixed in a solution of 2% paraformaldehyde in 100 mM potassium phosphate buffer pH = 7.0. 300 μL of fixation solution were added per well and two overnight incubations were done. After the incubations, the fixation solution was quenched with 0.1% of glycine (SIGMA) prepared in cell culture-grade PBS. Images of the cells were taken in light microscopy with the 10x objective lens, phase contrast and white correction. The photos were analyzed using the image processing software ImageJ from the National Institute of Health (USA). For each of the analyzed conditions, 1,000 cells were measured lengthwise and the information was recorded using a scale of 150 pixels = 50 μm, according to previous measurement of the scale bar with the image program. The data was analyzed with GraphPad Prism (GraphPad Software, USA) using a Kruskal-Wallis non-parametric test with *p* < 0.001.

## Supporting information

S1 Fig Alignment and amino acid sequence comparison of HldE proteins (bifunctional heptose 7-phosphate kinase/heptose 1-phosphate adenyltransferase; RfaE) in geographically diverse *H*. *pylori* strains. *H*. *pylori* strain designations, additional information and geographical origin as follows: B8—USA; J99—USA; P12—Germany; v225—South America; 26695—USA; B38 (*cag*PAI-negative strain from lymphoma patient)—France; F32—Japan; OK310 –Okinawa, Japan; SA_7—South Africa; N6 –France (the latter one is the reference strain in the present study). Nucleotide sequences were extracted and translated into amino acids from NCBI genome database entries using the software MEGA v4.1. The alignment was generated by ClustalW and depicted using GeneDoc software (http://www.nrbsc.org/old/gfx/genedoc/). Grey shading indicates the extent of conservation of the single residues. The line at the bottom of the alignment depicts the consensus sequence. The region around amino acid 325 of the sequences includes the hinge region between the two functional domains of the HldE protein. A strong inter-strain variability can be observed between the sequences originating from geographically diverse isolates both with and without the presence of a *cag*PAI. Accession numbers: N6 GCA_000285895.1; B8 GCA_000196755.1; J99 GCA_000982695.1; P12 GCA_000021465.1; 26695 GCA_000008525.1; B38 GCA_000091345.1; F32 GCA_000270045.1; OK310 GCA_000348885.1; SouthAfrica (SA_)7 GCA_000185245.1.
(TIF)

S2 Fig Characterization of *hldE* LPS inner core heptose gene cluster mutants in N6 and P12 *H*. *pylori* strains. **A), B), C), D)** Transmission EM images (Methods) of plate-grown *H*. *pylori* N6 wild type (wt) and mutants in the *hldE* LPS inner core heptose gene cluster and the *hldE* (HP0858) complemented strain. No major morphological differences were observed between the parental strains and mutants, and all bacterial strains exhibited characteristic flagellar bundles. *hldE* = HP0858, *rfaD* = HP0859. Size bars correspond to 2 μm. **E)** Antibiotic susceptibility testing by E-test for tetracyclin (Tet) and rifampicin (Rif) for *H*. *pylori* N6wt, its isogenic HP0858 mutant and HP0858-complemented (comp.) strain. MIC values in [mg/L] are indicated in the lower right corner of each image subpanel (for Tet) and in the summary table (for Tet and Rif) as mean and standard error of two measurements for each condition. **F)** Comparative Western blots of *H*. *pylori* whole bacterial lysates of parental strain N6, *cagY* (HP0527) mutant and mutants in the inner core heptose gene cluster as indicated (see main Fig 1), subsequently detecting CagA, other CagPAI proteins (CagM, CagL) and strain-specific outer membrane proteins (HopZII, BabB) with respective antisera, as indicated to the right of the panels. α-FlhA polyclonal antiserum [111] and α-catalase monoclonal antibody (R-Biopharm, Germany) were used to detect inner membrane protein and soluble cytoplasmic protein, respectively. A loading control (bottom panel) was performed using commercial antiserum against heat-inactivated *H*. *pylori* (Dako, Denmark). The same blot was re-used for all antisera and was stripped in between detection cycles. **G)** CagA and CagL were quantitated by densitometry from the blots summarized in panel E) (values were normalized to HP-specific band in bottom panel and are depicted on the y-axis in %, relative to N6 wild type control, which was set to 100%). **H)** Surface detection of fixed bacteria of strains N6 and P12 using anti-*H*. *pylori* surface-directed antiserum, providing a control for main Fig 1H. Plate-grown bacteria were fixed to gelatin-coated wells of a 96 well plate using 2% paraformaldehyde and detected using antiserum against heat-inactivated *H*. *pylori* surface antigens (Dako, Denmark). Statistics in H) was determined using Student’s *t*-test (** p<0.01; *** p<0.001; **** p<0.0001; ns is not significant).
(TIF)

S3 Fig Live-dead stain of different *H*. *pylori* LPS inner core heptose gene cluster mutants. Bacteria from pre-cultures grown either on plates or in liquid culture (mid-log phase) as indicated were stained for 5 min in liquid growth medium adjusted to an O.D._600_ of 0.1 using the Bac-Light Live/Dead Bacterial Viability Kit (Molecular Probes/ThermoFisher Scientific). Morphological differences of bacterial cells observed mainly upon growth in liquid culture included more variable bacterial length, shorter or more filamentous cells, or a more aggregative growth for the LPS heptose mutants. Plate-grown bacteria of all strains revealed no major morphological differences except for HP0859 and HP0860 mutants which tended to form shorter cell bodies. Bacteria were recorded at 40-fold lens magnification using an Olympus IX-40 inverted microscope in fluorescence mode.
(TIF)

S4 Fig Determining the role of LPS inner core heptose biosynthesis-derived signaling by *H*. *pylori* during interaction with human THP-1 cell line positive for TLR4/MD2-dependent LPS recognition. Adherent THP-1-luc cell line (monocyte-macrophage-like cells, differentiated state after lentiviral transduction using the Cignal Lenti NFkB Reporter (luciferase) system of SABiosciences/Qiagen;[112]) were coincubated with *H*. *pylori* N6, its isogenic core heptose mutants and the CagT4SS functional negative mutant *cagY* (HP0527) at an MOI of 25 bacteria per cell for 4 h. IL-8 release into the supernatants was quantitated by ELISA. LPS activation (control) is shown in the two bars to the right of the panel with an additional mock-coincubated control (100 ng ultrapure *E*. *coli* LPS [List Laboratories] was added to the cells for 3 h). The statistical significance of differences (biological duplicate experiments measured in triplicates) were determined by Students *t*-test (****p<0.001).
(TIF)

S5 Fig Filtered culture supernatants from bacterial liquid cultures do not exhibit any core heptose biosynthesis-dependent activation potential. In **A)** and **B)**, AGS cells were coincubated for 4 h with sterile-filtered *H*. *pylori* (N6) culture supernatants (collected from 24 h or 48 h bacteria liquid cultures), each aliquot equivalent to 10 μl from a bacterial liquid culture at O.D._600_ of 0.35 (from ca. 1x 10^8^ bacteria). Absolute values for released IL-8 are given. Live bacteria of *H*. *pylori* wild type strain 88–3887 (26695A; Live bac) were used as a control for positive cell activation (statistically different from mock; ****p<0.0001 [ANOVA; GraphPad]). All other conditions of supernatant coincubation were not statistically different from mock-coincubated cells. In panels **C)** and **D)**, HEK-Blue Null1 SEAP reporter cells were lipofectamine-transfected with bacterial sterile-filtered supernatants (from 24 h or 48 h grown liquid cultures, 10 μl of an O.D._600_ = 0.35, as above) and monitored for color change of the HEK Blue detection medium for up to 14 h after transfection (performed in technical duplicates). All values in C) and D) were low and in the range of or below mock-transfected values and indicate no activation.
(TIF)

S6 Fig Quantification of hummingbird phenotype in *H*. *pylori*-coincubated gastric epithelial cells / length distribution (addendum to main Fig 3). AGS cells were co-incubated with different strains of *H*. *pylori* at an MOI of 25 for 4 h. Cells were fixed and images captured at a lens magnification of 10-fold using an inverted Zeiss microscope (see main Fig 3). Images were evaluated for hummingbird phenotype (cell elongation) using the software ImageJ (Methods). Panels **A**) to **D**) and **F**), **G**) depict a length distribution graph of 1,000 single cells (unbiased cell selection, either AGS parent or TIFA CRISPR/Cas9 k/o cells) under each co-incubation condition as indicated above the panels, showing the percentage of cell lengths between 5 and 90 μm (maximum). Percentages for below and above 25 μm length of all cells under each condition are shown above each panel. The average cell width was 11.6 μm (±2.76 μm) for mock-infected and wild type bacteria-infected AGS wt cells (50 non-random cells counted for each condition). Panel **E**) depicts as a schematic how the cell length and width were measured in ImageJ. The size bar corresponds to 10 μm. I) IL-8 release by AGS cells coincubated with *H*. *pylori* 88–3887 wt, isogenic *cagA* or *cag*PAI mutants (4 h and 24 h coincubation time). Summary of biological triplicates measured in duplicates (t-test; ****p<0.0001).
(TIF)

S7 Fig Complementation of TIFA in AGS cells, detection of TIFA expression. AGS TIFA knock-out (KO) cells (cell pool) were transiently transfected with empty vector (Empty) or with TIFA expression plasmid (TIFA) (Supplementary S4 Table). Cells were mock coincubated or coincubated with live bacterial strains (as indicated; designations correspond to main text and main figures). Equal amounts of cleared lysates from the cells (20 μg) were loaded and separated on an SDS gel. Expression of TIFA was detected by reprobing the same Western blot membrane using TIFA antibody or anti-DDK antibody, which recognizes a Flag tag fused to TIFA as expressed by the complementation plasmid. Actin antibody detection of the same blot membrane served as a loading control. Non spec. band = designates a non-specific band of unknown identity recognized by the native TIFA antibody.
(TIF)

S8 Fig Relative qPCR array results for AGS wild type cells and TIFA k/o cells coincubated or not with *H*. *pylori* N6 wild type strain (Extended information to main Fig 4). RT^2^ Profiler RT-PCR arrays (Innate and Adaptive Immune Responses gene panel; Qiagen, Hilden, Germany) were performed on cDNAs of AGS cells mock-coincubated or coincubated with diverse *H*. *pylori* strains in order to screen for changed transcripts. Shown are scatter plots for pairwise comparisons generated by the online evaluation software RT^2^ Profiler Data Analysis v.3.5 (Qiagen, Hilden, Germany), and the respective gene tables for over- and under-regulated genes of the same pairwise comparisons, as fold change values (see also main Fig 4). **A**) shows group 1 (control group) (x-axis) versus group 2 (y-axis) pairwise comparison of fold changes of transcript (normalized log_10_ values); **B**) genes over- or under-expressed in group 2 vs. group 1. **C**) depicts group 3 (x-axis) versus group 1 (y-axis) pairwise comparison; **D**) genes over- or under-expressed in group 3 vs. group 1 (control group). Dashed lines in panels A) and C) represent the threshold of two-fold regulated. **E**) shows a real-time qPCR verification of nod1 transcript under different coincubation conditions. **F**) depicts the pairwise comparison scatter plot between group 4 (x-axis) and group 3 (y-axis). **G)** genes over-expressed in group 3 vs. group 4; and genes under-expressed in group 3 vs. group 4. **H**) shows the pairwise comparison plot between group 3 (x-axis) and group 5 (y-axis). **I**) genes over-expressed in group 5 vs. group 3; and genes under-expressed in group 5 vs. group 3. Dashed lines in panels F) and H) represent the threshold of two-fold regulated. **K**) Full results of RT^2^ Profiler RT-PCR arrays (Innate and Adaptive Immune Responses gene panel; Qiagen, Hilden, Germany), depicted as fold-regulation (in comparison to control group [= group 1]) of all detected genes under all conditions (group 2 through 5). Fold-regulation values of all tested genes for all experimental groups were combined in one bar graph. Experimental group 1: AGS wild type (wt) cells, mock-coincubated was used as control condition for the fold-regulation with a value of 0. Experimental group 1 (control group): AGS wild type (wt) cells, mock-coincubated; group 2: AGS TIFA CRISPR/Cas9 k/o cells, pool, (AGSTIFA), mock-coincubated; group 3: AGS wt cells, coincubated with *H*. *pylori* (HP) N6 parental strain (HPN6wt); group 4: AGS wt cells, coincubated with HP N6 *hldE* (HPN6 HP0858) mutant; group 5: AGS TIFA k/o cells, coincubated with HPN6wt strain. Diagonal black lines depict log_10_ normalized values for x and y axis comparison for each included transcript. Dashed lines represent the threshold of 2-fold for changed transcripts. Yellow dots represent over-expressed genes for condition (group) shown on y-axis. Blue dots represent under-expressed genes for condition (group) on y-axis. Selected transcripts were verified using standard qRT-PCR (Methods and main figures). For all experimental groups except group 3, two arrays were averaged. Exp. group 3 contains three averaged arrays. For detailed explanations of the array, gene designations and data evaluation, including normalization and thresholds for significance categories (Comments) of regulated transcripts, see S2 Table and online: www.qiagen.com.
(PDF)

S9 Fig Activation potential of *H*. *pylori* cell infection or transfection with bacterial enzymatically treated lysate (ETL) preparations monitored in HEK293 reporter cells. **A**) concentration-dependent activation of HEK293 NF-kB luciferase reporter cells by transfection with *H*. *pylori* ETL preparations generated from N6 wild type (wt) strain or N6 HP0858 (*hldE*) mutant. HEK293T cells were transiently transfected with a luciferase reporter plasmid (pNFkB-luc, BD Biosciences) carrying a firefly luciferase gene under the control of a NF-κB promotor (main Methods). Subsequently, transfected HEK293T cells were either mock-incubated or super-transfected with respective ETL preparations at the indicated amounts for 4 h. Each condition was measured in triplicates. While transfected N6 wt ETL activated reporter cells in a concentration-dependent manner, transfected N6 HP0858 mutant ETL did not significantly activate the reporter cells at any given concentration (*t*-test *p value< 0.05; **p<0.01). **B**), **C**), **D**), **E**): HEK-Blue Null1 SEAP reporter cells (Invivogen) were either co-incubated (**B**), **D**)) with live *H*. *pylori* strains, or (**C**), **E**)) transfected (main Methods) with ETL preparations of the respective strains as indicated in the legends. HEK-Blue Null1 reporter cells express secreted alkaline phosphatase (SEAP) under the control of multiple NF-kB and AP-1 binding sites. Each condition was measured in triplicates. The graphs show time-dependent cumulative activation of secreted alkaline phosphatase by the reporter cells over a time course of 25 h after transfection. Mock values were subtracted. ETL preparations added into the cell medium in the absence of transfection agent did not activate the reporter cells (see main Fig 5B). Panels **B**) and **C**) show comparative activation by infection **B**) or ETL transfection **C**) of HP N6 and its respective inner core heptose mutants; **D**) and **E**) show the activation potential after co-incubation with live bacteria (**D**) or transfections of ETL preparations **E**) from a selection of *H*. *pylori* wild type (wt) isolates. ETL from various *H*. *pylori* wt isolates showed differential activation potential upon transfection. Statistical significance of differences in B) and C) was calculated by Student’s *t*-test for comparisons between mock and all other conditions (blue symbols) and HPN6 wt and all other conditions (red symbols); ns = non-significant; **p<0.01; ***p<0.001; ****p<0.0001.
(TIF)

S1 Table Tukey’s multiple comparison tests^a^ for statistical significance p of growth as O.D._600_ at different time points between growth curves of *H*. *pylori* N6 wild type strain and different heptose mutants. (PDF)

S2 Table Complete results of RT^2^ Profiler PCR Array (Innate and Adaptive Immune Responses Panel, Qiagen PAHS-052Z), performed on cDNAs of AGS wild type cells and AGS TIFA knock/out cells under different coincubation conditions; see separate Excel file S2 Table. (XLSX)

S3 Table Bacterial strains used in this study. (PDF)

S4 Table Plasmids used in this study. (PDF)

S5 Table Primers for cloning, PCR and sequencing (origin: this study). (PDF)

S6 Table Antibodies used for detection of proteins. (PDF)

S1 References (PDF)
