# Supplementary figures and images for: Helicobacter pylori modulates host cell responses by CagT4SS-dependent translocation of an intermediate metabolite of LPS inner core heptose biosynthesis
Source: PLoS Pathog. 2017 Jul 17;13(7):e1006514. doi: 10.1371/journal.ppat.1006514 (PMC5531669; doi:10.1371/journal.ppat.1006514)

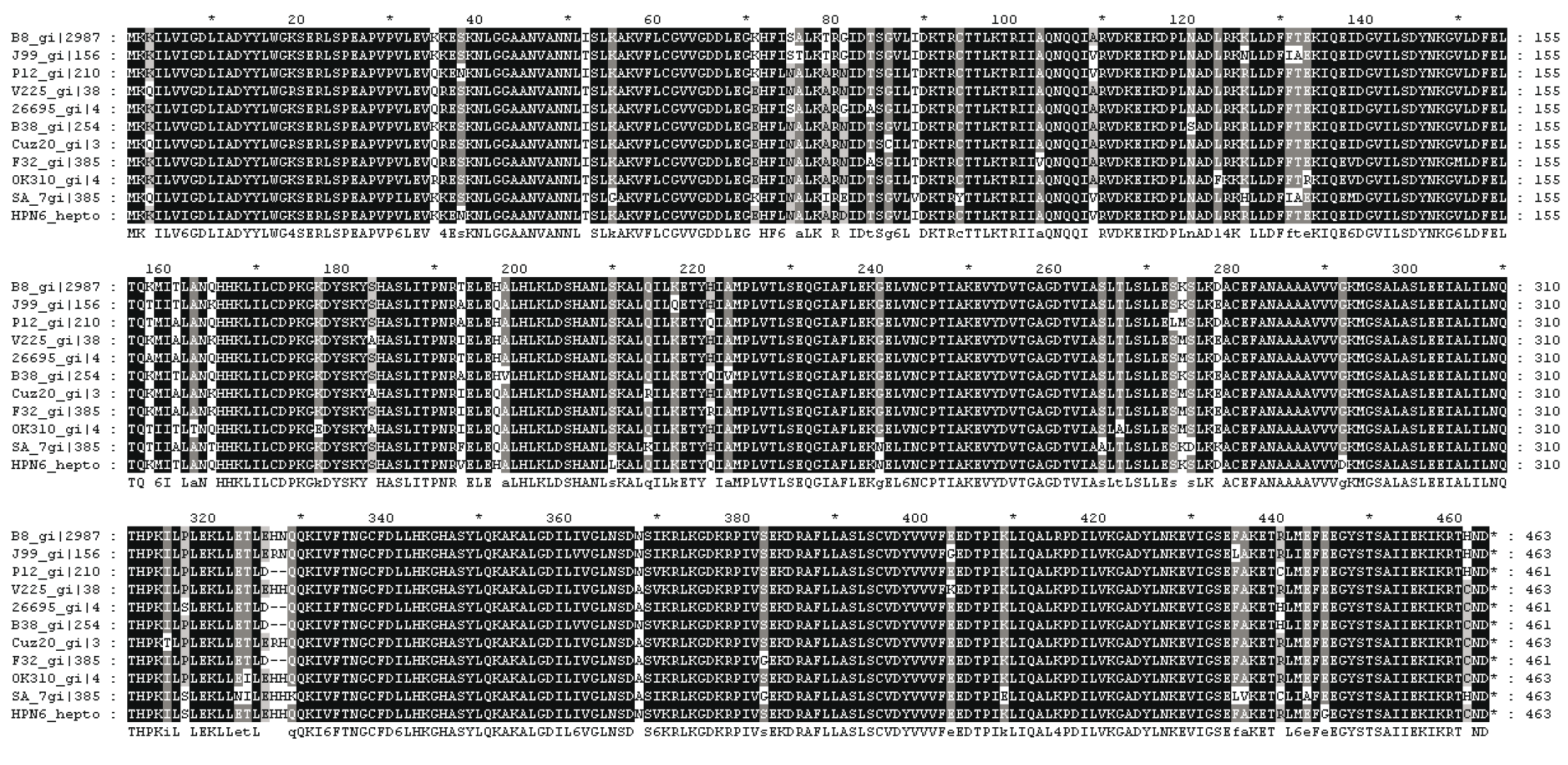

Supplement: S1 Fig — H. pylori strain designations, additional information and geographical origin as follows: B8—USA; J99—USA; P12—Germany; v225—South America; 26695—USA; B38 (cagPAI-negative strain from lymphoma patient)—France; F32—Japan; OK310 –Okinawa, Japan; SA_7—South Africa; N6 –France (the latter one is the reference strain in the present study). Nucleotide sequences were extracted and translated into amino acids from NCBI genome database entries using the software MEGA v4.1. The alignment was generated by ClustalW and depicted using GeneDoc software (http://www.nrbsc.org/old/gfx/genedoc/). Grey shading indicates the extent of conservation of the single residues. The line at the bottom of the alignment depicts the consensus sequence. The region around amino acid 325 of the sequences includes the hinge region between the two functional domains of the HldE protein. A strong inter-strain variability can be observed between the sequences originating from geographically diverse isolates both with and without the presence of a cagPAI. Accession numbers: N6 GCA_000285895.1; B8 GCA_000196755.1; J99 GCA_000982695.1; P12 GCA_000021465.1; 26695 GCA_000008525.1; B38 GCA_000091345.1; F32 GCA_000270045.1; OK310 GCA_000348885.1; SouthAfrica (SA_)7 GCA_000185245.1. (TIF) [file ppat.1006514.s001.tif]

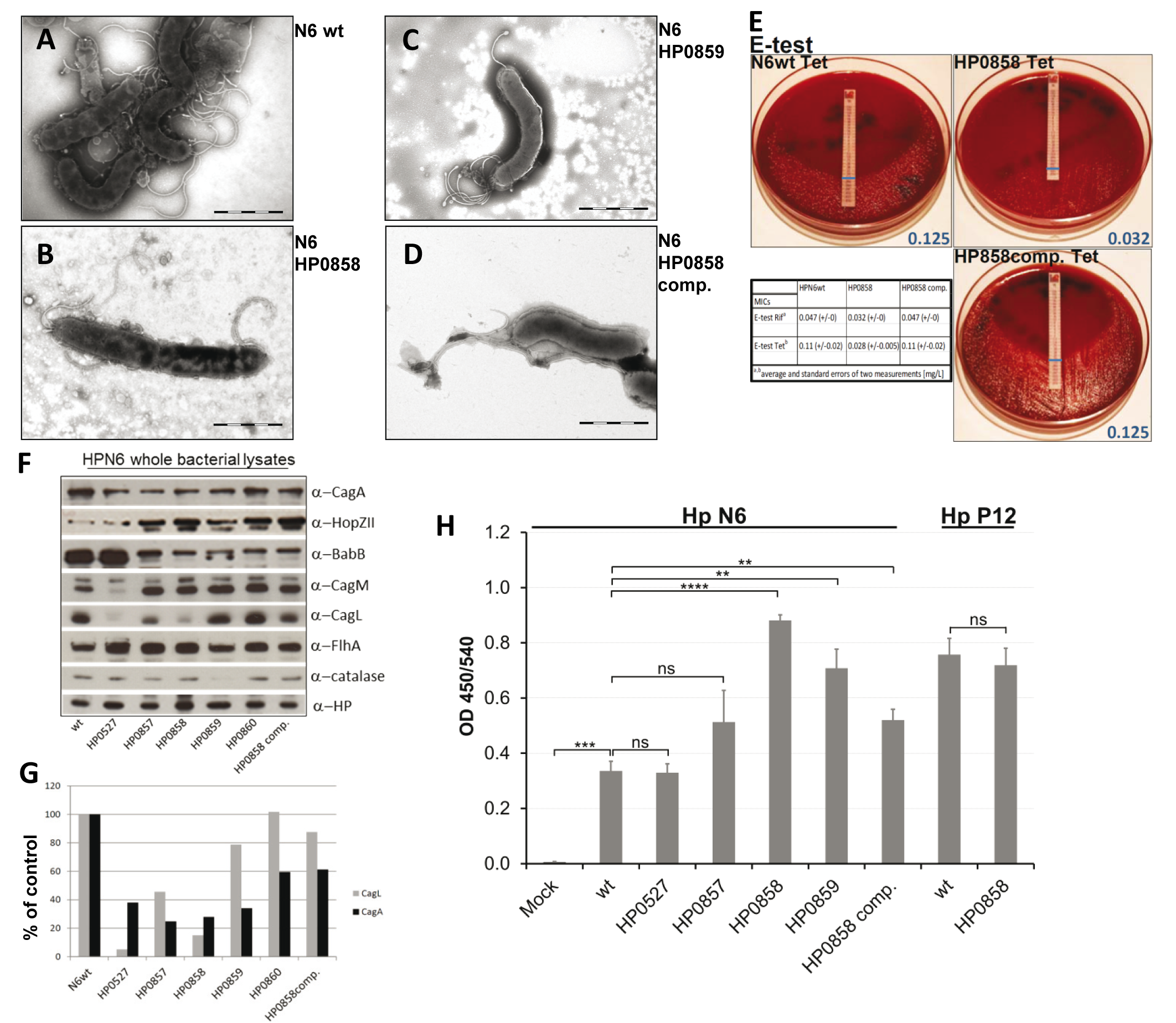

Supplement: S2 Fig — A), B), C), D) Transmission EM images (Methods) of plate-grown H. pylori N6 wild type (wt) and mutants in the hldE LPS inner core heptose gene cluster and the hldE (HP0858) complemented strain. No major morphological differences were observed between the parental strains and mutants, and all bacterial strains exhibited characteristic flagellar bundles. hldE = HP0858, rfaD = HP0859. Size bars correspond to 2 μm. E) Antibiotic susceptibility testing by E-test for tetracyclin (Tet) and rifampicin (Rif) for H. pylori N6wt, its isogenic HP0858 mutant and HP0858-complemented (comp.) strain. MIC values in [mg/L] are indicated in the lower right corner of each image subpanel (for Tet) and in the summary table (for Tet and Rif) as mean and standard error of two measurements for each condition. F) Comparative Western blots of H. pylori whole bacterial lysates of parental strain N6, cagY (HP0527) mutant and mutants in the inner core heptose gene cluster as indicated (see main Fig 1), subsequently detecting CagA, other CagPAI proteins (CagM, CagL) and strain-specific outer membrane proteins (HopZII, BabB) with respective antisera, as indicated to the right of the panels. α-FlhA polyclonal antiserum [111] and α-catalase monoclonal antibody (R-Biopharm, Germany) were used to detect inner membrane protein and soluble cytoplasmic protein, respectively. A loading control (bottom panel) was performed using commercial antiserum against heat-inactivated H. pylori (Dako, Denmark). The same blot was re-used for all antisera and was stripped in between detection cycles. G) CagA and CagL were quantitated by densitometry from the blots summarized in panel E) (values were normalized to HP-specific band in bottom panel and are depicted on the y-axis in %, relative to N6 wild type control, which was set to 100%). H) Surface detection of fixed bacteria of strains N6 and P12 using anti-H. pylori surface-directed antiserum, providing a control for main Fig 1H. Plate-grown bacteria were fixed to g [file ppat.1006514.s002.tif]

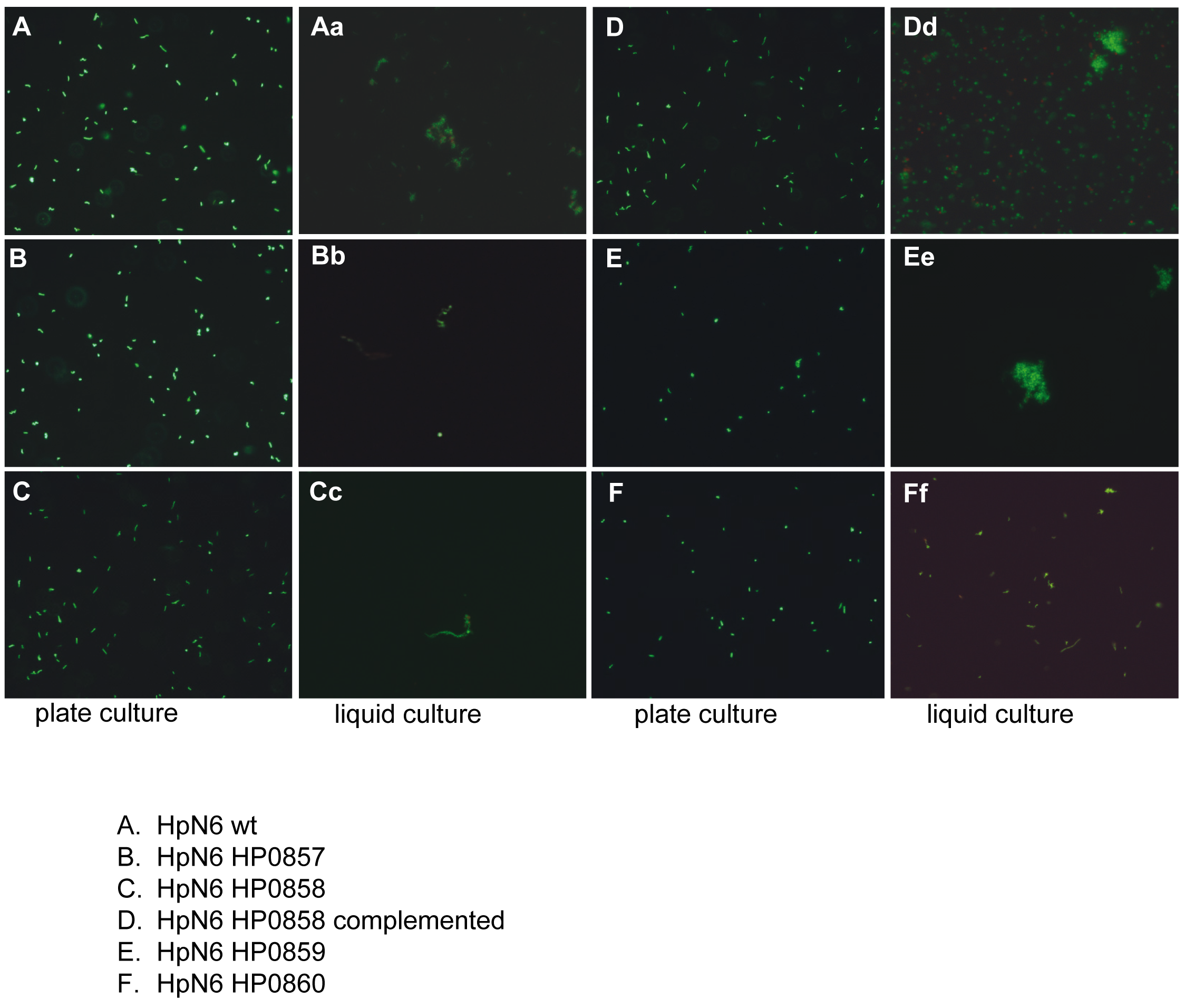

Supplement: S3 Fig — Bacteria from pre-cultures grown either on plates or in liquid culture (mid-log phase) as indicated were stained for 5 min in liquid growth medium adjusted to an O.D.600 of 0.1 using the Bac-Light Live/Dead Bacterial Viability Kit (Molecular Probes/ThermoFisher Scientific). Morphological differences of bacterial cells observed mainly upon growth in liquid culture included more variable bacterial length, shorter or more filamentous cells, or a more aggregative growth for the LPS heptose mutants. Plate-grown bacteria of all strains revealed no major morphological differences except for HP0859 and HP0860 mutants which tended to form shorter cell bodies. Bacteria were recorded at 40-fold lens magnification using an Olympus IX-40 inverted microscope in fluorescence mode. (TIF) [file ppat.1006514.s003.tif]

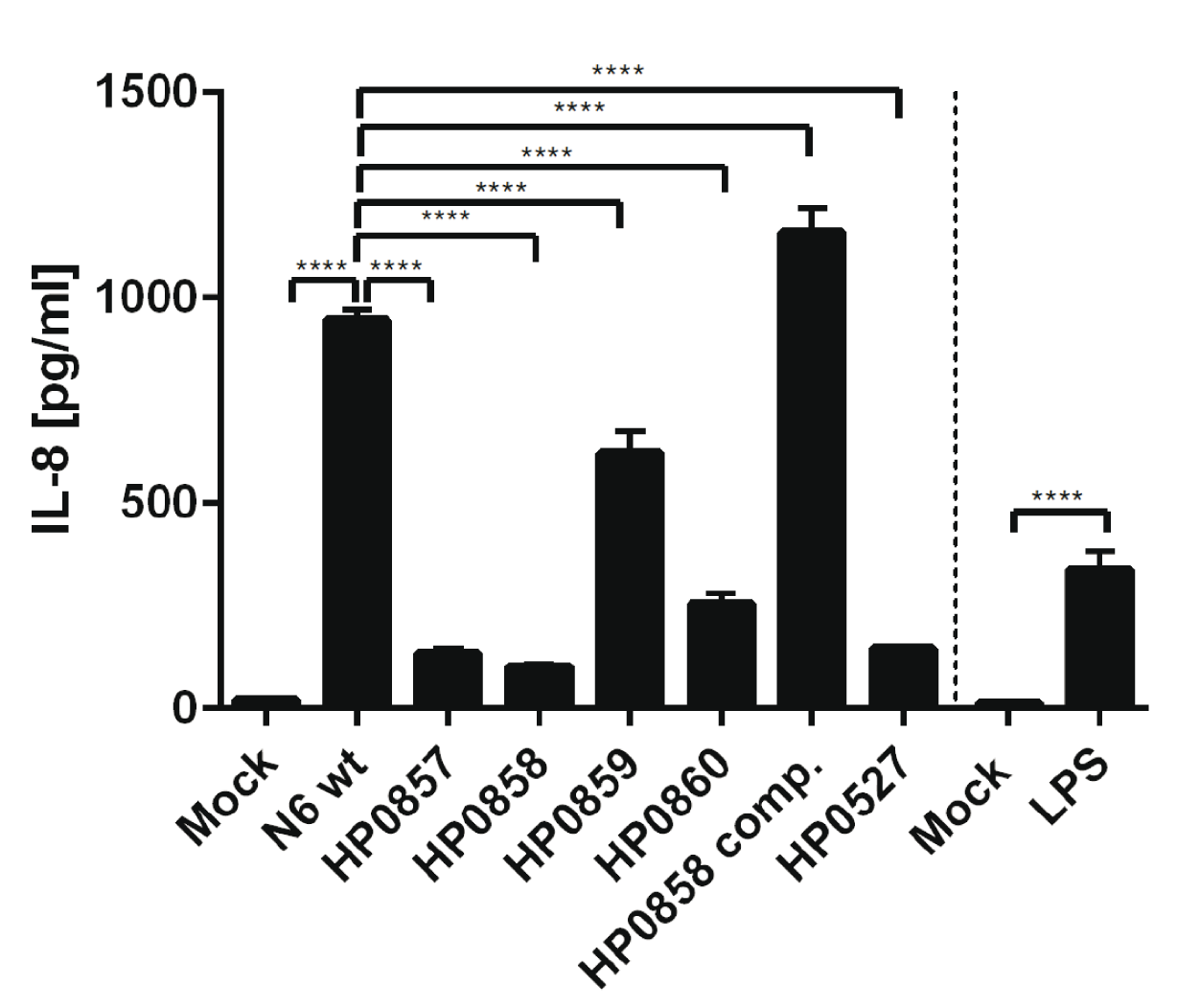

Supplement: S4 Fig — Adherent THP-1-luc cell line (monocyte-macrophage-like cells, differentiated state after lentiviral transduction using the Cignal Lenti NFkB Reporter (luciferase) system of SABiosciences/Qiagen;[112]) were coincubated with H. pylori N6, its isogenic core heptose mutants and the CagT4SS functional negative mutant cagY (HP0527) at an MOI of 25 bacteria per cell for 4 h. IL-8 release into the supernatants was quantitated by ELISA. LPS activation (control) is shown in the two bars to the right of the panel with an additional mock-coincubated control (100 ng ultrapure E. coli LPS [List Laboratories] was added to the cells for 3 h). The statistical significance of differences (biological duplicate experiments measured in triplicates) were determined by Students t-test (****p<0.001). (TIF) [file ppat.1006514.s004.tif]

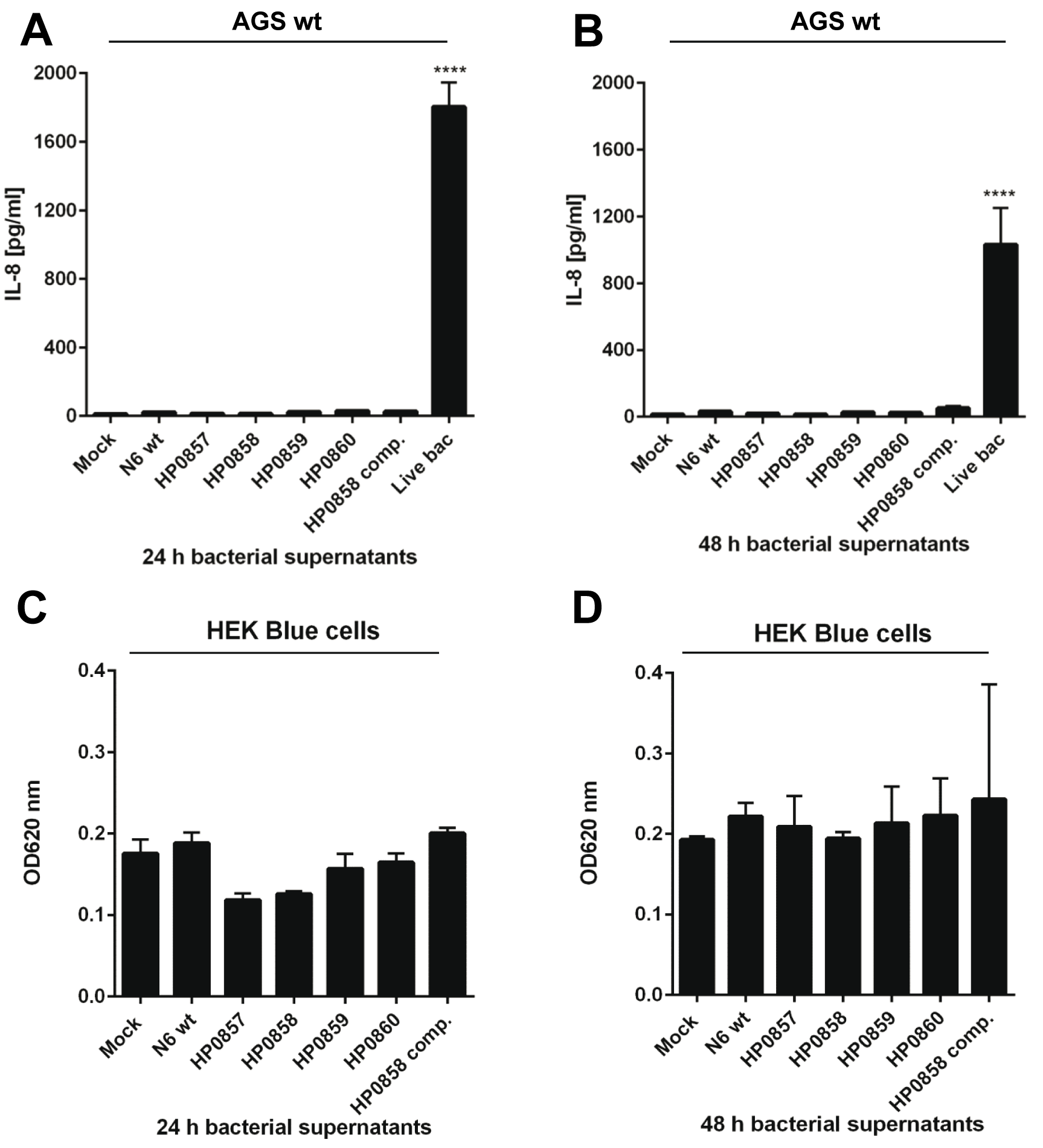

Supplement: S5 Fig — In A) and B), AGS cells were coincubated for 4 h with sterile-filtered H. pylori (N6) culture supernatants (collected from 24 h or 48 h bacteria liquid cultures), each aliquot equivalent to 10 μl from a bacterial liquid culture at O.D.600 of 0.35 (from ca. 1x 10^8^ bacteria). Absolute values for released IL-8 are given. Live bacteria of H. pylori wild type strain 88–3887 (26695A; Live bac) were used as a control for positive cell activation (statistically different from mock; ****p<0.0001 [ANOVA; GraphPad]). All other conditions of supernatant coincubation were not statistically different from mock-coincubated cells. In panels C) and D), HEK-Blue Null1 SEAP reporter cells were lipofectamine-transfected with bacterial sterile-filtered supernatants (from 24 h or 48 h grown liquid cultures, 10 μl of an O.D.600 = 0.35, as above) and monitored for color change of the HEK Blue detection medium for up to 14 h after transfection (performed in technical duplicates). All values in C) and D) were low and in the range of or below mock-transfected values and indicate no activation. (TIF) [file ppat.1006514.s005.tif]

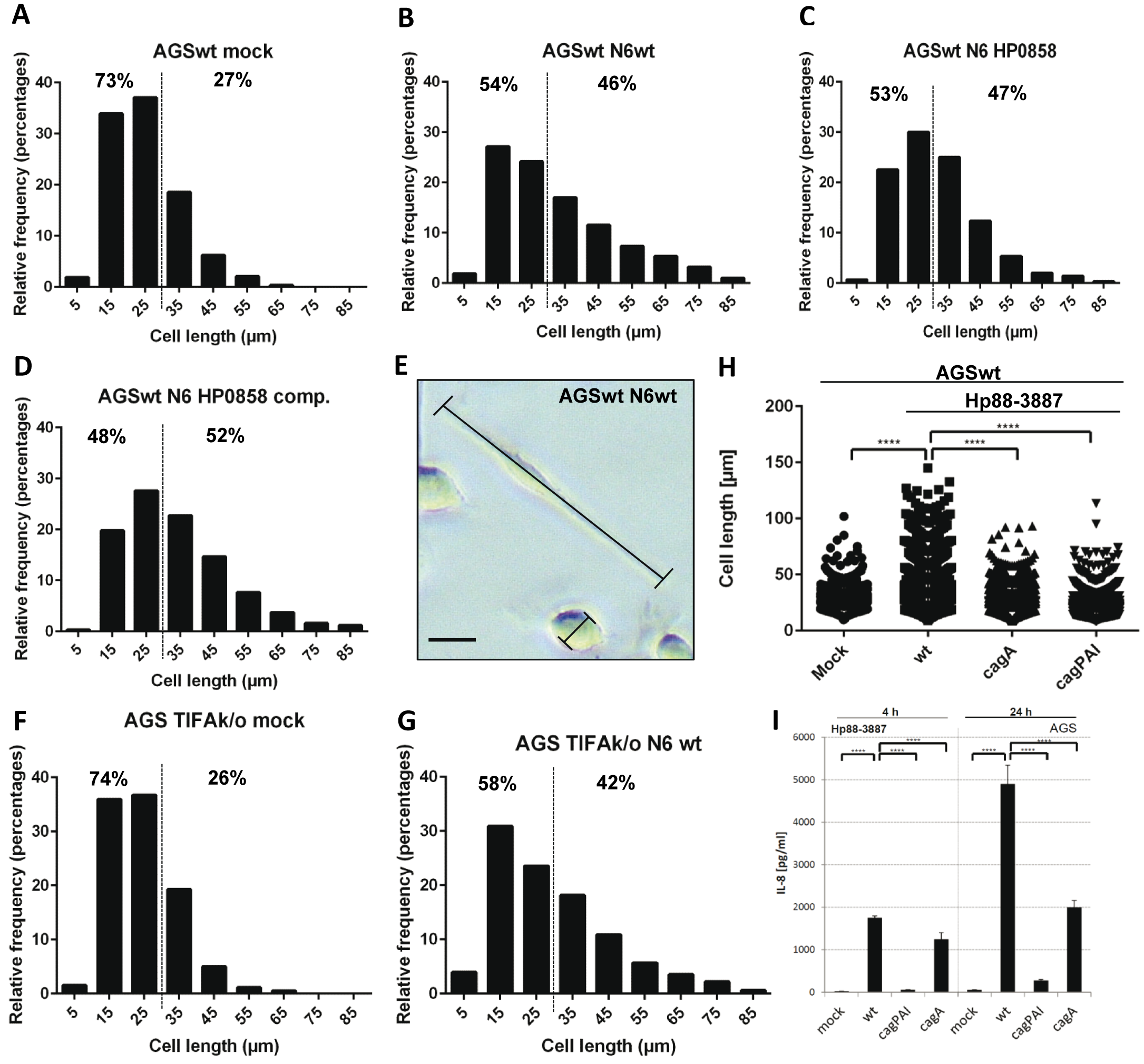

Supplement: S6 Fig — AGS cells were co-incubated with different strains of H. pylori at an MOI of 25 for 4 h. Cells were fixed and images captured at a lens magnification of 10-fold using an inverted Zeiss microscope (see main Fig 3). Images were evaluated for hummingbird phenotype (cell elongation) using the software ImageJ (Methods). Panels A) to D) and F), G) depict a length distribution graph of 1,000 single cells (unbiased cell selection, either AGS parent or TIFA CRISPR/Cas9 k/o cells) under each co-incubation condition as indicated above the panels, showing the percentage of cell lengths between 5 and 90 μm (maximum). Percentages for below and above 25 μm length of all cells under each condition are shown above each panel. The average cell width was 11.6 μm (±2.76 μm) for mock-infected and wild type bacteria-infected AGS wt cells (50 non-random cells counted for each condition). Panel E) depicts as a schematic how the cell length and width were measured in ImageJ. The size bar corresponds to 10 μm. I) IL-8 release by AGS cells coincubated with H. pylori 88–3887 wt, isogenic cagA or cagPAI mutants (4 h and 24 h coincubation time). Summary of biological triplicates measured in duplicates (t-test; ****p<0.0001). (TIF) [file ppat.1006514.s006.tif]

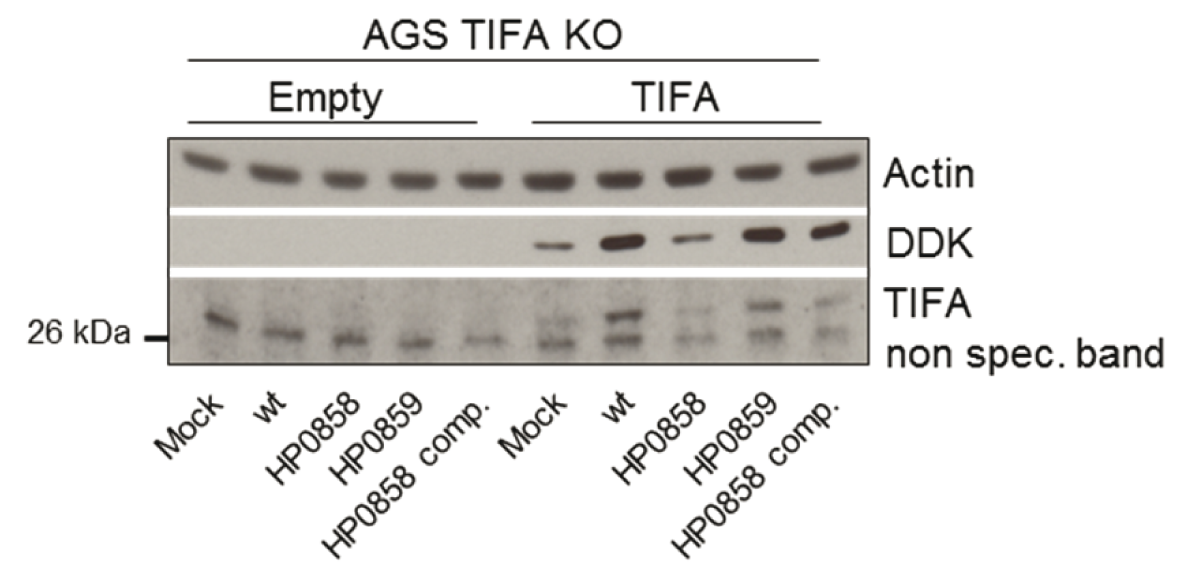

Supplement: S7 Fig — AGS TIFA knock-out (KO) cells (cell pool) were transiently transfected with empty vector (Empty) or with TIFA expression plasmid (TIFA) (Supplementary S4 Table). Cells were mock coincubated or coincubated with live bacterial strains (as indicated; designations correspond to main text and main figures). Equal amounts of cleared lysates from the cells (20 μg) were loaded and separated on an SDS gel. Expression of TIFA was detected by reprobing the same Western blot membrane using TIFA antibody or anti-DDK antibody, which recognizes a Flag tag fused to TIFA as expressed by the complementation plasmid. Actin antibody detection of the same blot membrane served as a loading control. Non spec. band = designates a non-specific band of unknown identity recognized by the native TIFA antibody. (TIF) [file ppat.1006514.s007.tif]

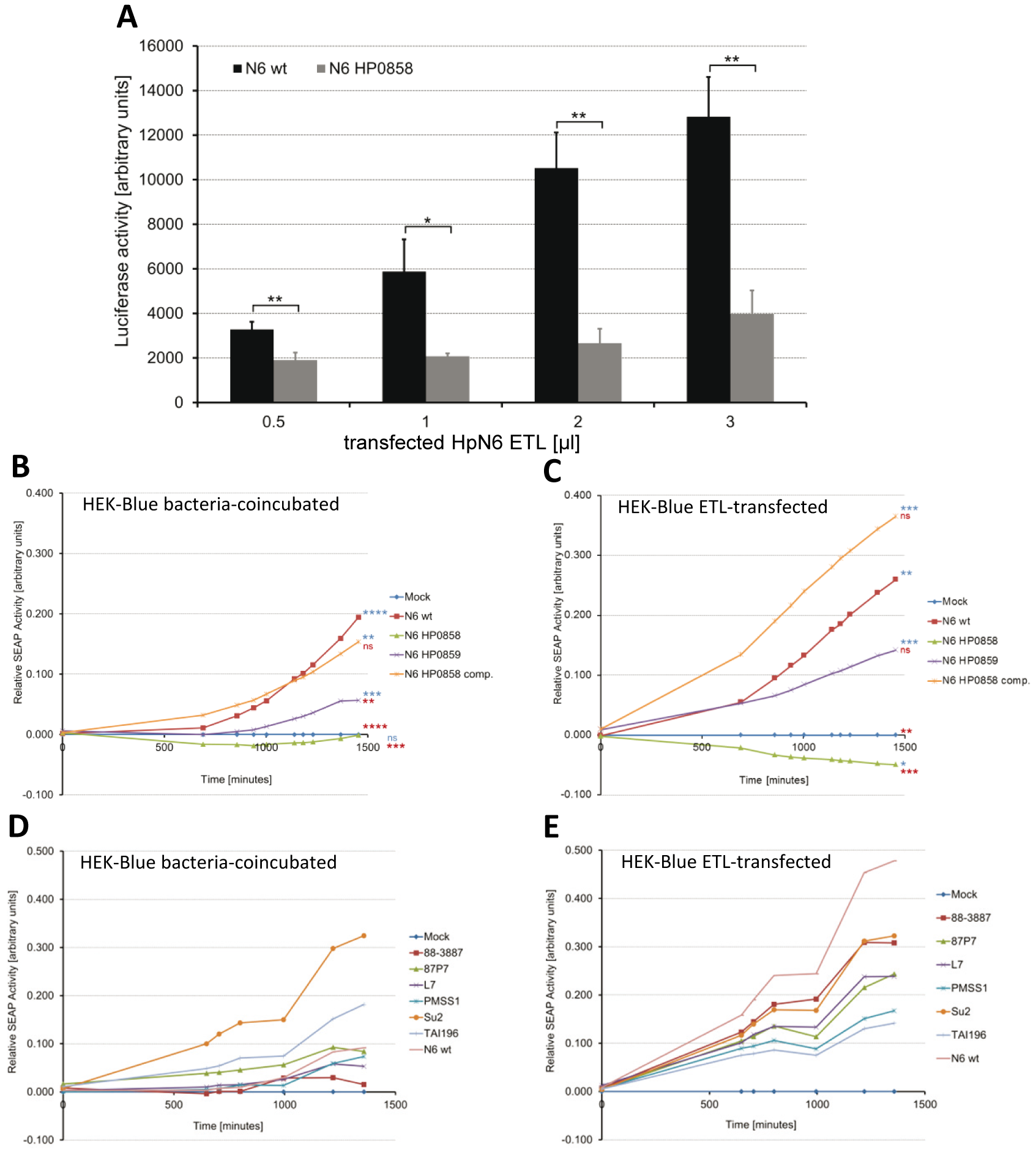

Supplement: S9 Fig — A) concentration-dependent activation of HEK293 NF-kB luciferase reporter cells by transfection with H. pylori ETL preparations generated from N6 wild type (wt) strain or N6 HP0858 (hldE) mutant. HEK293T cells were transiently transfected with a luciferase reporter plasmid (pNFkB-luc, BD Biosciences) carrying a firefly luciferase gene under the control of a NF-κB promotor (main Methods). Subsequently, transfected HEK293T cells were either mock-incubated or super-transfected with respective ETL preparations at the indicated amounts for 4 h. Each condition was measured in triplicates. While transfected N6 wt ETL activated reporter cells in a concentration-dependent manner, transfected N6 HP0858 mutant ETL did not significantly activate the reporter cells at any given concentration (t-test *p value< 0.05; **p<0.01). B), C), D), E): HEK-Blue Null1 SEAP reporter cells (Invivogen) were either co-incubated (B), D)) with live H. pylori strains, or (C), E)) transfected (main Methods) with ETL preparations of the respective strains as indicated in the legends. HEK-Blue Null1 reporter cells express secreted alkaline phosphatase (SEAP) under the control of multiple NF-kB and AP-1 binding sites. Each condition was measured in triplicates. The graphs show time-dependent cumulative activation of secreted alkaline phosphatase by the reporter cells over a time course of 25 h after transfection. Mock values were subtracted. ETL preparations added into the cell medium in the absence of transfection agent did not activate the reporter cells (see main Fig 5B). Panels B) and C) show comparative activation by infection B) or ETL transfection C) of HP N6 and its respective inner core heptose mutants; D) and E) show the activation potential after co-incubation with live bacteria (D) or transfections of ETL preparations E) from a selection of H. pylori wild type (wt) isolates. ETL from various H. pylori wt isolates showed differential activation potential upon transfection. Statistical signi [file ppat.1006514.s009.tif]
