# Supplementary material for: Helicobacter pylori modulates host cell responses by CagT4SS-dependent translocation of an intermediate metabolite of LPS inner core heptose biosynthesis
Source: PLoS Pathog. 2017 Jul 17;13(7):e1006514. doi: 10.1371/journal.ppat.1006514 (PMC5531669; doi:10.1371/journal.ppat.1006514)

A

AGSTIFA mock (2)/AGSwt mock (1; control)

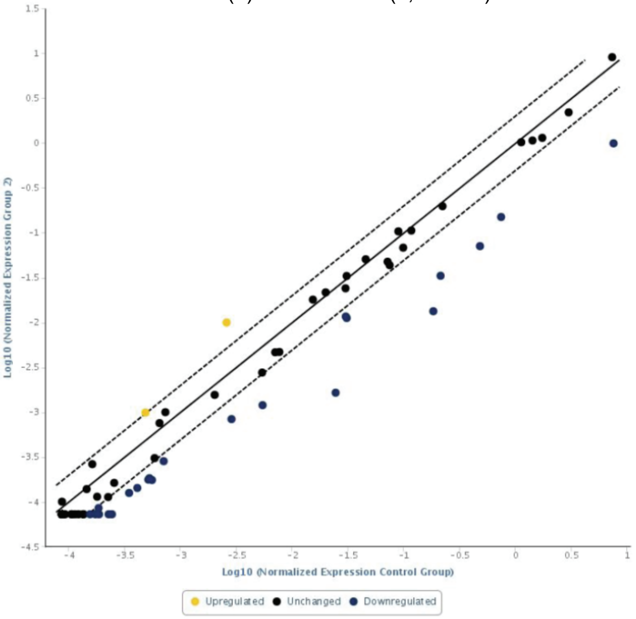

C

AGSwt HPN6wt (3)/AGSwt mock (1; control)

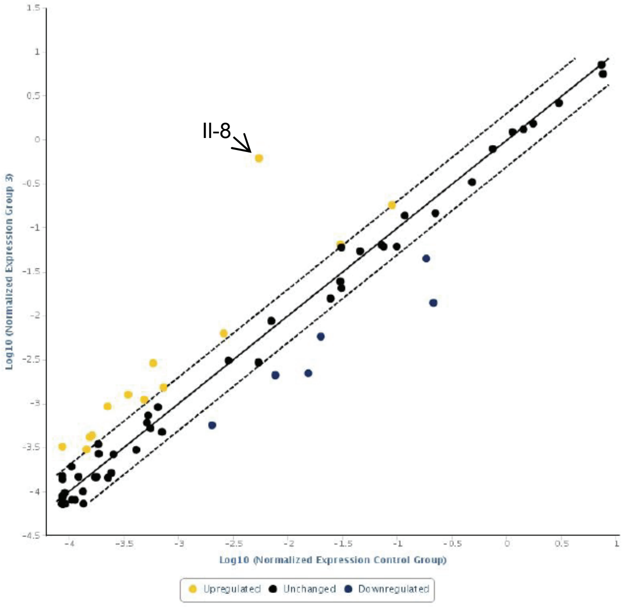

B

Genes Over-Expressed in Group 2 vs. Control Group

Position	Gene Symbol	Fold Regulation	Comments	RT2 Catalog
A12	CD40	3.88		PPH00296D
E06	MAPK8	2.05		PPH00720B

Genes Under-Expressed in Group 2 vs. Control Group

Position	Gene Symbol	Fold Regulation	Comments	RT2 Catalog
C01	HLA-A	-14.78		PPH05978F
C12	IL18	-13.73		PPH00580C
H01	ACTB	-7.65		PPH00073G
E04	LYZ	-6.78		PPH14748A
C02	HLA-E	-6.42		PPH18633B
H02	B2M	-4.96		PPH01094E
D09	CXCL8	-4.51		PPH00568A
F02	NOD1	-3.40	A	PPH00891C
D01	IL1A	-3.31		PPH00690A
A05	CCL5	-3.14		PPH00703B
F04	RAG1	-3.09		PPH09892A
C06	IFNB1	-2.89		PPH00384F
D02	IL1B	-2.84		PPH00171C
B07	CXCL10	-2.84		PPH00765E
F10	STAT6	-2.74		PPH00760D
C04	IFNA1	-2.73		PPH01321B
C03	ICAM1	-2.58		PPH00640F
C09	IL10	-2.54		PPH00572C
G03	TLR3	-2.46		PPH01803E
F06	SLC11A1	-2.35		PPH05732F
E02	JAK2	-2.14		PPH00758C
E01	ITGAM	-2.11		PPH00644F

D

Genes Over-Expressed in Group 3 vs. Control Group

Position	Gene Symbol	Fold Regulation	Comments	RT2 Catalog
D09	CXCL8	113.64		PPH00568A
D05	IL23A	4.91	A	PPH01688B
B06	CSF2	4.13		PPH00576C
G10	TNF	3.75		PPH00341F
C04	IFNA1	3.65	A	PPH01321B
E01	ITGAM	2.70		PPH00644F
B11	FOXP3	2.70		PPH00029C
A12	CD40	2.43		PPH00296D
E06	MAPK8	2.30		PPH00720B
C03	ICAM1	2.12		PPH00640F
B09	DDX58	2.09		PPH20774A
F08	STAT3	2.09	A	PPH00708F
E12	NFKBIA	2.02		PPH00170F

Genes Under-Expressed in Group 3 vs. Control Group

Position	Gene Symbol	Fold Regulation	Comments	RT2 Catalog
C02	HLA-E	-15.25		PPH18633B
G12	TYK2	-6.90		PPH00775A
C12	IL18	-4.12		PPH00580C
G11	TRAF6	-3.64		PPH00329B
C05	IFNAR1	-3.56	A	PPH00869F
F12	TICAM1	-3.45		PPH06044A

E

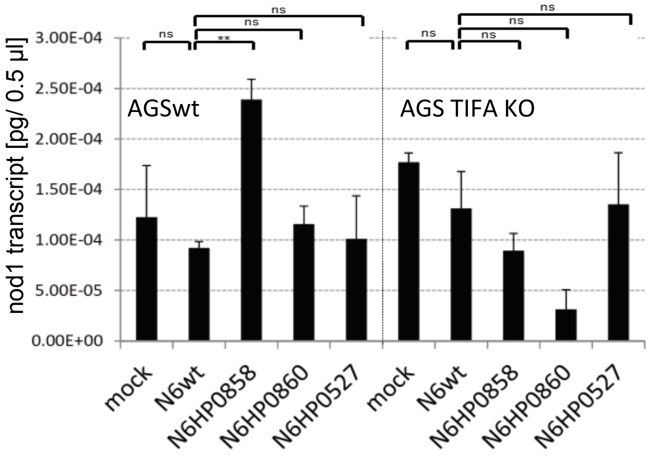

F

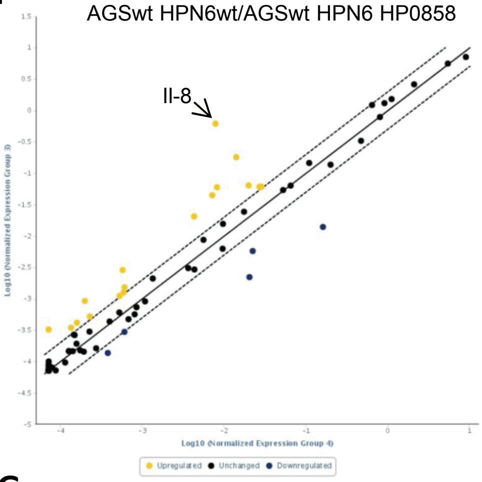

H

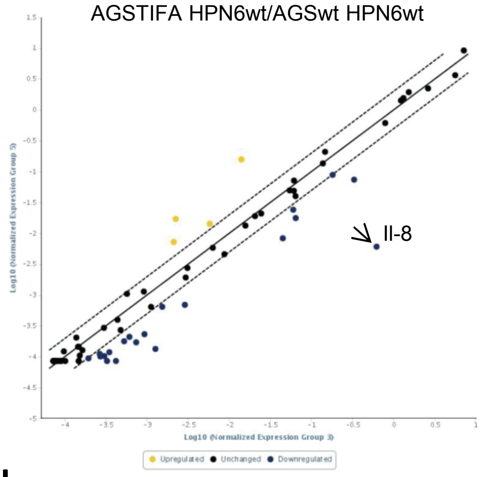

G

Genes Over-Expressed in Group 3 vs. Group 4

Position	Gene Symbol	Fold Regulation	Comments	RT2 Catalog
D09	CXCL8	78.46		PPH00568A
E12	NFKBIA	12.76		PPH00170F
E11	NFKB1	7.29		PPH00204F
C12	IL18	6.22		PPH00580C
D05	IL23A	4.99	A	PPH01688B
F10	STAT6	4.75		PPH00760D
B06	CSF2	4.65		PPH00576C
G10	TNF	4.51		PPH00341F
C03	ICAM1	3.21		PPH00640F
E01	ITGAM	2.61		PPH00644F
E02	JAK2	2.56		PPH00758C
F08	STAT3	2.51	A	PPH00708F
A05	CCL5	2.28		PPH00703B
D10	IRAK1	2.25		PPH00835A
C04	IFNA1	2.13	A	PPH01321B
E10	MYD88	2.12		PPH00911B
E06	MAPK8	2.10		PPH00720B

Genes Under-Expressed in Group 3 vs. Group 4

Position	Gene Symbol	Fold Regulation	Comments	RT2 Catalog
C02	HLA-E	-11.57		PPH18633B
G12	TYK2	-9.22		PPH00775A
F12	TICAM1	-3.88		PPH06044A
A11	CD4	-2.77		PPH01629C
D02	IL1B	-2.04		PPH00171C

I

Genes Over-Expressed in Group 5 vs. Group 3

Position	Gene Symbol	Fold Regulation	Comments	RT2 Catalog
C02	HLA-E	11.24		PPH18633B
G12	TYK2	7.71		PPH00775A
G11	TRAF6	3.43		PPH00329B
F12	TICAM1	2.46		PPH06044A

Genes Under-Expressed in Group 5 vs. Group 3

Position	Gene Symbol	Fold Regulation	Comments	RT2 Catalog
D09	CXCL8	-101.50		PPH00568A
C04	IFNA1	-9.40	A	PPH01321B
C12	IL18	-5.37		PPH00580C
E01	ITGAM	-4.86		PPH00644F
E04	LYZ	-4.45		PPH14748A
B07	CXCL10	-4.27		PPH00765E
D05	IL23A	-4.13	A	PPH01688B
B06	CSF2	-3.98		PPH00576C
G10	TNF	-3.78		PPH00341F
C03	ICAM1	-3.63		PPH00640F
A05	CCL5	-2.93		PPH00703B
B09	DDX58	-2.93		PPH20774A
E02	JAK2	-2.92		PPH00758C
C06	IFNB1	-2.89		PPH00384F
C09	IL10	-2.66		PPH00572C
E11	NFKB1	-2.48		PPH00204F
G06	TLR6	-2.37		PPH01798E
F08	STAT3	-2.36	A	PPH00708F
B08	CXCR3	-2.04		PPH01041A
E12	NFKBIA	-2.04		PPH00170F

K fold-regulation of transcripts (RT^2 qRT-PCR array) of all conditions (groups) in comparison to control condition (control group 1): AGSwt cells, mock-coincubated)

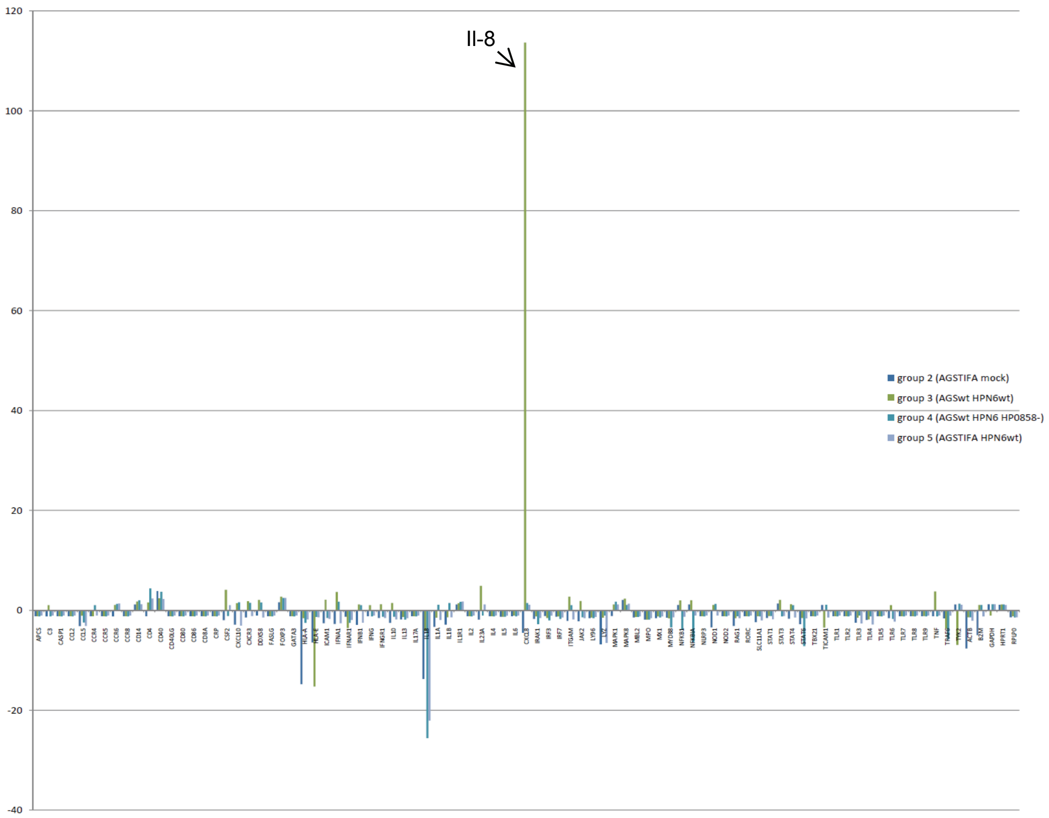

Supplement: S8 Fig — RT2 Profiler RT-PCR arrays (Innate and Adaptive Immune Responses gene panel; Qiagen, Hilden, Germany) were performed on cDNAs of AGS cells mock-coincubated or coincubated with diverse H. pylori strains in order to screen for changed transcripts. Shown are scatter plots for pairwise comparisons generated by the online evaluation software RT2 Profiler Data Analysis v.3.5 (Qiagen, Hilden, Germany), and the respective gene tables for over- and under-regulated genes of the same pairwise comparisons, as fold change values (see also main Fig 4). A) shows group 1 (control group) (x-axis) versus group 2 (y-axis) pairwise comparison of fold changes of transcript (normalized log10 values); B) genes over- or under-expressed in group 2 vs. group 1. C) depicts group 3 (x-axis) versus group 1 (y-axis) pairwise comparison; D) genes over- or under-expressed in group 3 vs. group 1 (control group). Dashed lines in panels A) and C) represent the threshold of two-fold regulated. E) shows a real-time qPCR verification of nod1 transcript under different coincubation conditions. F) depicts the pairwise comparison scatter plot between group 4 (x-axis) and group 3 (y-axis). G) genes over-expressed in group 3 vs. group 4; and genes under-expressed in group 3 vs. group 4. H) shows the pairwise comparison plot between group 3 (x-axis) and group 5 (y-axis). I) genes over-expressed in group 5 vs. group 3; and genes under-expressed in group 5 vs. group 3. Dashed lines in panels F) and H) represent the threshold of two-fold regulated. K) Full results of RT2 Profiler RT-PCR arrays (Innate and Adaptive Immune Responses gene panel; Qiagen, Hilden, Germany), depicted as fold-regulation (in comparison to control group [= group 1]) of all detected genes under all conditions (group 2 through 5). Fold-regulation values of all tested genes for all experimental groups were combined in one bar graph. Experimental group 1: AGS wild type (wt) cells, mock-coincubated was used as control condition for the fold-re [file ppat.1006514.s008.pdf]
