# Supplementary material for: Helicobacter pylori modulates host cell responses by CagT4SS-dependent translocation of an intermediate metabolite of LPS inner core heptose biosynthesis
Source: PLoS Pathog. 2017 Jul 17;13(7):e1006514. doi: 10.1371/journal.ppat.1006514 (PMC5531669; doi:10.1371/journal.ppat.1006514)
Supplement: S1 Table — (PDF) [file ppat.1006514.s010.pdf]

Number of families (growth curves)	8		
Number of comparisons per family	15		
Alpha	0.05		
**Tukey's multiple comparisons test**	**Mean difference**	**95% CI[b] of difference**	**Significant[c]**
**Row 1 (T=0 h)[d]**			
N6 wt vs. HP0857	0.0	-0.3619 to 0.3619	ns
N6 wt vs. HP0858	0.0	-0.3619 to 0.3619	ns
N6 wt vs. HP0859	0.0	-0.3619 to 0.3619	ns
N6 wt vs. HP0860	0.0	-0.3619 to 0.3619	ns
N6 wt vs. HP0858 comp.	0.0	-0.3619 to 0.3619	ns
**Row 2 (T=15 h)**			
N6 wt vs. HP0857	0.3285	-0.03338 to 0.6904	ns
N6 wt vs. HP0858	0.2985	-0.06338 to 0.6604	ns
N6 wt vs. HP0859	0.2045	-0.1574 to 0.5664	ns
N6 wt vs. HP0860	0.2540	-0.1079 to 0.6159	ns
N6 wt vs. HP0858 comp.	-0.1115	-0.4734 to 0.2504	ns
**Row 3 (T=19 h)**			
N6 wt vs. HP0857	0.5545	0.1113 to 0.9977	**
N6 wt vs. HP0858	0.5160	0.07279 to 0.9592	*
N6 wt vs. HP0859	0.3710	-0.07221 to 0.8142	ns
N6 wt vs. HP0860	0.4490	0.005792 to 0.8922	*
N6 wt vs. HP0858 comp.	-0.1205	-0.5637 to 0.3227	ns
HP0857 vs. HP0858	-0.0385	-0.4004 to 0.3234	ns
**Row 4 (T=21 h)**			
N6 wt vs. HP0857	0.6770	0.3151 to 1.039	****
N6 wt vs. HP0858	0.6285	0.2666 to 0.9904	****
N6 wt vs. HP0859	0.3910	0.02912 to 0.7529	*
N6 wt vs. HP0860	0.5385	0.1766 to 0.9004	***
N6 wt vs. HP0858 comp.	-0.0785	-0.4404 to 0.2834	ns
**Row 5 (T=23 h)**			
N6 wt vs. HP0857	0.8065	0.4446 to 1.168	****
N6 wt vs. HP0858	0.7475	0.3856 to 1.109	****
N6 wt vs. HP0859	0.4665	0.1046 to 0.8284	**
N6 wt vs. HP0860	0.6375	0.2756 to 0.9994	****
N6 wt vs. HP0858 comp.	-0.1675	-0.5294 to 0.1944	ns
**Row 6 (T=25 h)**			
N6 wt vs. HP0857	0.9545	0.5926 to 1.316	****
N6 wt vs. HP0858	0.8995	0.5376 to 1.261	****
N6 wt vs. HP0859	0.5375	0.1756 to 0.8994	***
N6 wt vs. HP0860	0.7680	0.4061 to 1.130	****
N6 wt vs. HP0858 comp.	-0.1140	-0.4759 to 0.2479	ns
**Row 7 (T=39 h)**			
N6 wt vs. HP0857	2.560	2.198 to 2.921	****
N6 wt vs. HP0858	2.350	1.988 to 2.711	****
N6 wt vs. HP0859	0.2570	-0.1049 to 0.6189	ns
N6 wt vs. HP0860	1.460	1.098 to 1.821	****
N6 wt vs. HP0858 comp.	0.09500	-0.2669 to 0.4569	ns
**Row 8 (T=48 h)**			
N6 wt vs. HP0857	2.772	2.410 to 3.133	****
N6 wt vs. HP0858	2.544	2.182 to 2.906	****
N6 wt vs. HP0859	-0.7770	-1.139 to -0.4151	****
N6 wt vs. HP0860	1.147	0.7851 to 1.509	****
N6 wt vs. HP0858 comp.	0.5480	0.1861 to 0.9099	***

[a] performed on the basis of a Two-Way ANOVA

[b]CI = confidence interval

[c]significances:*p<0.05; **p<0.01;***p<0.001;****p<0.0001

[d] time points (T) of measurement (hours)
