# Supplementary material for: Helicobacter pylori modulates host cell responses by CagT4SS-dependent translocation of an intermediate metabolite of LPS inner core heptose biosynthesis
Source: PLoS Pathog. 2017 Jul 17;13(7):e1006514. doi: 10.1371/journal.ppat.1006514 (PMC5531669; doi:10.1371/journal.ppat.1006514)
Supplement: S3 Table — (PDF) [file ppat.1006514.s012.pdf]

Strain name	Species	Description	Origin
Hp N6	*H. pylori*	Wild type strain N6, human isolate (France)	[113;114]
Hp N6 HP0527 (*cagY*)	*H. pylori*	HP0527 (*cagY*) allelic exchange insertion mutant in strain N6[a]	This study
Hp N6 HP0857 (*gmhA*)	*H. pylori*	HP0857 (*gmhA*) allelic exchange insertion mutant in strain N6[a]	This study
Hp N6 HP0858 (*hldE*)	*H. pylori*	HP0858 (*hldE*) allelic exchange insertion mutant in strain N6[a]	This study
Hp N6 HP0859 (*rfaD*)	*H. pylori*	HP0859 (*rfaD*/*hldD*) allelic exchange insertion mutant in strain N6[a]	This study
Hp N6 HP0860 (*gmhB*)	*H. pylori*	HP0860 (*gmhB*) allelic exchange insertion mutant in strain N6[a]	This study
Hp N6 HP0858 comp.	*H. pylori*	HP0858 complementation in *rdxA* locus of HP0858 knockout, strain N6[a]	This study
Hp P12	*H. pylori*	Wild type strain P12 (Germany)	[115]
Hp P12 HP0858 (*hldE*)	*H. pylori*	HP0858 (*hldE*) allelic exchange insertion mutant in strain P12[a]	This study
Hp 88-3887 (HP26695A)	*H. pylori*	Human isolate (USA), low passage number	[116;8]
Hp 88-3887 Δ*cag*PAI	*H. pylori*	*cag*PAI deletion by allelic exchange in strain 88-3887 (plasmid pCJ324)	[117]
Hp 88-3887 *cagA*	*H. pylori*	*cagA* allelic exchange insertion mutant in strain 88-3887	[117]

[a] insertion mutants were generated using flanking PCR products from plasmids containing insertions of a kanamycin or chloramphenicol cassette in each of the genes. Respective plasmids including antibiotic resistance information are listed in Supplementary Table S4.
