# Supplementary material for: Helicobacter pylori modulates host cell responses by CagT4SS-dependent translocation of an intermediate metabolite of LPS inner core heptose biosynthesis
Source: PLoS Pathog. 2017 Jul 17;13(7):e1006514. doi: 10.1371/journal.ppat.1006514 (PMC5531669; doi:10.1371/journal.ppat.1006514)
Supplement: S4 Table — (PDF) [file ppat.1006514.s013.pdf]

Plasmid name	Description	Function	Origin/reference
KN204357D	Origene CRISPR Cas9 KN204357	Human TIFA Knockout	Origene
KN204357G1/G2	Origene CRISPR Cas9 KN204357	Human TIFA Knockout	Origene
pCMV6_TIFA_Hs	Origene True ORF Gold, RC204357	hTIFA expression	Origene
pEF-BOS	Eukaryotic expression plasmid	Vector (cloning backbone)	[119]
pEF6-V5-empty	Eukaryotic expression plasmid	Empty vector control	[120]
pNFκB-luc	Contains firefly luciferase gene from *Photinus pyralis.* Expression is controlled by multiple NF-κB consensus sequences fused to a TATA-like promoter (P_{TAL}) region from the Herpes simplex virus thymidine kinase (HSV-TK) promoter. (PT3244-5, Cat. #6053-1).	NF-κB reporter plasmid expressing Luciferase	BD Biosciences
pCJ1624	HP0858 under control of the CagM promoter (both from strain *H. pylori* 26695) framed by *rdxA* arms of homology in pCJ542 backbone [108].	HP0858 complementation in *H. pylori*	This study
pCJ1625	pEF-BOS-empty with kanamycin cassette (from pILL600, [106]) framed by HP0859 (Hp 26695) arms of homology in multiple cloning site.	HP0859 insertion mutant	This study
pCJ1626	pUC18 with kanamycin cassette (pILL600, [106]) framed by HP0860 (Hp 26695) arms of homology in multiple cloning site.	HP0860 insertion mutant	This study
pCJ1627	pET28a with HP0857 sequence (Hp 26695) in frame with His-tag.	HP0857 cloning	This study
pCJ1629	pCJ1627 with CAT (Chloramphenicol acetyl transferase) cassette (pBHpC8, [121]) integrated in HP0857 (Hp 26695) sequence.	HP0857 insertion mutant	This study
