# Supplementary material for: Helicobacter pylori modulates host cell responses by CagT4SS-dependent translocation of an intermediate metabolite of LPS inner core heptose biosynthesis
Source: PLoS Pathog. 2017 Jul 17;13(7):e1006514. doi: 10.1371/journal.ppat.1006514 (PMC5531669; doi:10.1371/journal.ppat.1006514)
Supplement: S5 Table — (PDF) [file ppat.1006514.s014.pdf]

Gene	Function	Name	Direction	Sequence[a]
HP0527 (*cagY*)	Cloning (km insertion), PCR	hp527_fw2	forward	TTAATTGCCACCTTTGGGGC
		Hp527_rv2	reverse	TATCATATGATTAGCTAAGGCTTTAGGTGG
		hp527_ fw1b	forward	TATGGATCCTCACGATAAGAACAGCGAC
		Hp527_rv1	reverse	ATGAATGAAGAAAACGATAAAC
HP0857 (*gmhA*)	Cloning (cm insertion), PCR	HP0857ko_fw	forward	AAAGGATCCAGTGTTTGCCAGACAAGTGG
		HP0857ko_KpnI_rv	reverse	ATAGGTACCCATAATCGTTCGCAATGGCG
	Expression cloning	HP0857xp_BamHI_fw	forward	TATGGATCCATTGATAATTTAATTAAAAAAGAATTTTTAGCCCAT
		HP0857xp_NotI_rv	reverse	TATGCGGCCGCCTAATTTTTATGAGCGAAATGCCTTTC
HP0858 (*hldE*)	Cloning (km insertion), PCR	Hp0858ko_fw1	forward	CGAGCGTGAAAGCGATGAAG
		Hp0858ko_NotI_rv1	reverse	TATGCGGCCGCCGCTAATGAAATGCTCGCCC
		Hp0858ko_BamHI_fw2	forward	AAAGGATCCCCGTTGCACCACGCTTAAAA
		Hp0858ko_rv2	reverse	GCCTGCCCCAGTTACATCAT
	Expression cloning	HP0858xp_BamHI_fw	forward	TATGGATCCAAAAAAATCTTAGTCATAGGCGATCTGA
		HP0858xp_NotI_rv	reverse	TATGCGGCCGCTCAATCATTGCATGTCCTTTTAATTTTTTCTA
	Complementation (insertion in *rdxA*)	Hp0858SpeIF	forward	TATACTAGTAGGATTATGAAAAAAATCTTAGTCATAGGCGATC
		HP0858XhoIR	reverse	TATCTCGAGTCAATCATTGCATGTCCTTTTAATTTTTTC
HP0859 (*rfaD*)	Cloning (km insertion), PCR	HP0859ko_fw1	forward	TATTCTAGACGCCCCAGAAGAAAATTGCG
		HP0859ko_NotI_rv1	reverse	TATGCGGCCGCTCAGCTGCAATCACCTCACC
		HP0859ko_BamHI_fw2	forward	AAAGGATCCCCGCTGTCTCTGATACGACC
		HP0859ko_rv2	reverse	TATTCTAGATGGCTTGGGAATAACCCACA
HP0860 (*gmhB*)	Cloning (km insertion), PCR	HP0860ko_fw2	forward	TATGAATTCTCTTAGGCGTGTTGAACGGG
		HP0860ko_NotI_rv1	reverse	TATGCGGCCGCGATTTCGCATGCTTTAGCAATTCA
		HP0860ko_BamHI_fw2	forward	AAAGGATCCCTGGGATCAACCGAGGCTAT
		HP0860ko_rv2	reverse	TATTCTAGAAGCTTTAGGGTGGTTCTCTTGA
aphA3-III	Kanamycin (km) cassette *aphA3-III* (pILL600, [106]), conferring kanamycin resistance	Km8_for_NotI	forward	TATGCGGCCGCAGCGAACCATTTGAGGTG
		Km9_rev_BamHI	reverse	TATGGATCCATCATCGATAAGCTTTTTAGAC
cat	Chloramphenicol acetyl transferase CAT (cm, [121]) cassette (pBHpC8), conferring chloramphenicol resistance	pCAT1_SpeI	reverse	CGCACTAGTAACAGCTATGACCATGATTACG
		pCAT2-KpnI	forward	AATGGTACCGATATCGCATGCCTGCAGAG
hTIFA	analytical PCR for CRISPR-Cas9 knockout of hTIFA	TIFA_int_left_f2	forward	TGATACCAGTGCAGGCTGTG
		TIFA_int_left_r	reverse	GTGATGCGGCACTCGATCTC
		Tifa-cDNA_r	reverse	GGATGGTAAACCGTCATCTGGA

[a] restriction sites are underlined in nucleotide sequence
