# Supplementary material for: Helicobacter pylori modulates host cell responses by CagT4SS-dependent translocation of an intermediate metabolite of LPS inner core heptose biosynthesis
Source: PLoS Pathog. 2017 Jul 17;13(7):e1006514. doi: 10.1371/journal.ppat.1006514 (PMC5531669; doi:10.1371/journal.ppat.1006514)
Supplement: S6 Table — (PDF) [file ppat.1006514.s015.pdf]

Antigen	Species	Dilution used for Western Blot	Diluent[a]	Catalog number	Manufacturer/reference
Phospho-CagA (p-EPIYA Peptide)	Rabbit (polyclonal)	1:333	5% skim milk in TBS-T	–	[31] (custom-produced)
CagA (*H. pylori*)	Rabbit (polyclonal)	1:5,000	5% skim milk in TBS-T	HPM-5001-5	Austral Biologicals (San Ramon, CA; USA)
Helicobacter pylori, heat-inactivated total bacteria	Rabbit (polyclonal)	1:5,000	5% skim milk in TBS-T	B0471	DakoCytomation
Actin	Mouse (monoclonal)	1:10,000	5% skim milk in TBS-T	sc-8432	Santa Cruz
Phospho-TAK1 (P-TAK1) (Thr184/187) (*Homo sapiens*)	Rabbit (polyclonal)	1:500	5% BSA in TBS-T	#4508	Cell Signaling
TAK1 (*Homo sapiens*)	Rabbit (polyclonal)	1:1,000	5% BSA in TBS-T	#4505	Cell Signaling
Phospho-IκBα (*Homo sapiens*)	Rabbit (monoclonal)	1:1,000	5% BSA in TBS-T	#2859	Cell Signaling
IκBα (L35A5) (*Homo sapiens*)	Mouse (monoclonal)	1:1,000	5% BSA in TBS-T	#4814	Cell Signaling
Phospho-p38 MAPK (P-p38)(Thr180/Tyr182)	Rabbit (polyclonal)	1:1,000	5% BSA in TBS-T	#9211	Cell Signaling
P38 MAPK	Rabbit (polyclonal)	1:1,000	5% BSA in TBS-T	#9212	Cell Signaling
HopZ II (*H. pylori*)	Rabbit (polyclonal)	1:5,000	5% skim milk in TBS-T	–	Custom-produced (Kennemann, Suerbaum unpublished); kindly provided by Sebastian Suerbaum
BabB (*H. pylori*)	Rabbit (polyclonal)	1:5,000	5% skim milk in TBS-T	–	Custom-produced (Nell, Suerbaum et al., unpublished); kindly provided by Sebastian Suerbaum
CagM (*H. pylori*)	Rabbit (polyclonal)	1:10,000	5% skim milk in TBS-T	–	Custom-produced (Josenhans, unpublished)
CagL (*H. pylori*)	Rabbit (polyclonal)	1:10,000	5% skim milk in TBS-T	–	[122]; kindly provided by Wolfgang Fischer
FlhA (*H. pylori*)	Rabbit (polyclonal)	1:5,000	5% skim milk in TBS-T	–	[111]
Catalase (*H. pylori*)	(monoclonal)	1:100	5% skim milk in TBS-T	from RidaScreen Femtolab HP stool antigen test	R-Biopharm, Darmstadt, Germany
DDK	Mouse (monoclonal)	1:1,000	5% skim milk in TBS-T	TA150015	OriGene
TIFA (*Homo sapiens*)	Rabbit (polyclonal)	1:1,000	5% BSA in TBS-T	PA5-26591	Thermo Scientific

[a]TBS-T (Tris-buffered saline with 0.1% Tween-20)
