# Supplementary material for: Helicobacter pylori modulates host cell responses by CagT4SS-dependent translocation of an intermediate metabolite of LPS inner core heptose biosynthesis
Source: PLoS Pathog. 2017 Jul 17;13(7):e1006514. doi: 10.1371/journal.ppat.1006514 (PMC5531669; doi:10.1371/journal.ppat.1006514)
Supplement: S1 References — (PDF) [file ppat.1006514.s016.pdf]

Supporting References

113. Ferrero RL, Cussac V, Courcoux P, Labigne A. Construction of isogenic urease-negative mutants of *Helicobacter pylori* by allelic exchange. J Bacteriol 1992; 174:4212-4217.

114. Behrens W, Bönig T, Suerbaum S, Josenhans C. Genome sequence of *Helicobacter pylori* hpEurope strain N6. J Bacteriol 2012; 194(14):3725-3726.

115. Fischer W, Windhager L, Rohrer S, Zeiller M, Karnholz A, Hoffmann R et al. Strain-specific genes of *Helicobacter pylori*: genome evolution driven by a novel type IV secretion system and genomic island transfer. Nucleic Acids Res 2010; 38(18):6089-6101.

116. Josenhans C, Eaton KA, Thevenot T, Suerbaum S. Switching of flagellar motility in *Helicobacter pylori* by reversible length variation of a short homopolymeric sequence repeat in *fliP*, a gene encoding a basal body protein. Infect Immun 2000; 68(8):4598-4603.

117. Coombs N, Sompallae R, Olbermann P, Gastaldello S, Goppel D, Masucci MG et al. *Helicobacter pylori* affects the cellular deubiquitinase USP7 and ubiquitin-regulated components TRAF6 and the tumour suppressor p53. Int J Med Microbiol 2011; 301(3):213-224.

118. Lee A, O'Rourke J, De Ungria MC, Robertson B, Daskalopoulos G, Dixon MF. A standardized mouse model of *Helicobacter pylori* infection: Introducing the Sydney strain. Gastroenterology 1997; 112:1386-1397.

119. Mizushima S, Nagata S. pEF-BOS, a powerful mammalian expression vector. Nucleic Acids Res 1990; 18(17):5322.

120. Lee SK, Stack A, Katzowitsch E, Aizawa SI, Suerbaum S, Josenhans C. *Helicobacter pylori* flagellins have very low intrinsic activity to stimulate human gastric epithelial cells via TLR5. Microbes Infect 2003; 5(15):1345-1356.

121. Ge Z, Hiratsuka K, Taylor DE. Nucleotide sequence and mutational analysis indicate that two *Helicobacter pylori* genes encode a P-type ATPase and a cation-binding protein associated with copper transport. Mol Microbiol 1995; 15:97-106.

122. Kutter S, Buhrdorf R, Haas J, Schneider-Brachert W, Haas R, Fischer W. Protein subassemblies of the *Helicobacter pylori* Cag type IV secretion system revealed by localization and interaction studies. J Bacteriol 2008; 190(6):2161-2171.
